# An Alternative Host Material for Long‐Lifespan Blue Organic Light‐Emitting Diodes Using Thermally Activated Delayed Fluorescence

**DOI:** 10.1002/advs.201600502

**Published:** 2017-03-23

**Authors:** Soo‐Ghang Ihn, Namheon Lee, Soon Ok Jeon, Myungsun Sim, Hosuk Kang, Yongsik Jung, Dal Ho Huh, Young Mok Son, Sae Youn Lee, Masaki Numata, Hiroshi Miyazaki, Rafael Gómez‐Bombarelli, Jorge Aguilera‐Iparraguirre, Timothy Hirzel, Alán Aspuru‐Guzik, Sunghan Kim, Sangyoon Lee

**Affiliations:** ^1^ Samsung Advanced Institute of Technology Samsung Electronics Co., LTD 130 Samsung‐ro, Yeongtong‐gu Suwon‐si Gyeonggi‐do 16678 South Korea; ^2^ Samsung SDI 130 Samsung‐ro, Yeongtong‐gu Suwon‐si Gyeonggi‐do 16678 South Korea; ^3^ Department of Chemistry and Chemical Biology Harvard University 12 Oxford St Cambridge MA 02138 USA

**Keywords:** blue, device lifespan, hosts, OLEDs, TADF

## Abstract

It has been challenging to find stable blue organic light emitting diodes (OLEDs) that rely on thermally activated delayed fluorescence (TADF). Lack of stable host materials well‐fitted to the TADF emitters is one of the critical reasons. The most popular host for blue TADF, bis[2‐(diphenylphosphino)phenyl] ether oxide (DPEPO), leads to unrealistically high maximum external quantum efficiency. DPEPO is however an unstable material and has a poor charge transporting ability, which in turn induces an intrinsic short OLED operating lifespan. Here, an alternative host material is introduced which educes the potential efficiency and device lifespan of given TADF emitters with the appropriateness of replacing the most popular host material, DPEPO, in developing blue TADF emitters. It simultaneously provides much longer device lifespan and higher external quantum efficiency at a practical brightness due to its high material stability and electron‐transport‐type character well‐fitted for hole‐transport‐type TADF emitters.

Blue organic light emitting diodes (OLEDs) can play an important role in displays.^1^ Efficient and stable blue organic electroluminescence (EL) is essential to meet the requirements of the display standard for the success of OLED technology. However, searching for efficient and stable blue organic emitters is still a great challenge. Blue organic emitters show inferior EL performance to the other primary‐color emitters because they need to have wider energy band gap (*E*_g_).^2^, ^3^ Thermally activated delayed fluorescence (TADF) emitters^4^, ^5^, ^6^ have thus been established because they are alternatives to phosphorescent emitters harvesting triplet states. In TADF emitters, nonemissive triplet states are harvested via repopulation of the emissive singlet state through reverse intersystem crossing (RISC) induced by ambient thermal energy due to the small energy gap (Δ*E*_ST_) between the lowest singlet (*S*_1_) and triplet (*T*_1_) states. 100% internal quantum efficiency (IQE) has already been realized^7^ and a blue TADF OLED has exhibited 30% maximum external quantum efficiency (EQE_max_).^8^ However, despite the significant progress in efficiency, it is unusual to find stable blue TADF OLEDs. Although various blue TADF emitters have been reported so far,^4^, ^5^, ^8^, ^9^, ^10^, ^11^, ^12^ there have been only a few articles claiming device lifespan. Kim et al. reported a blue TADF OLED exhibiting lifespan up to 80% (LT80) of initial luminance (500 cd m^−2^) of 52 h using 9,9′,9″,9′′′‐((6‐phenyl‐1,3,5‐triazine‐2,4‐diyl)bis(benzene‐5,1,3‐triyl))tetrakis(9H‐carbazole) (DDCzTrz).^10^ However, it exhibited a low EQE, probably lower than 2% at 500 cd m^−2^ which is much lower than those of traditional fluorescent OLEDs (≈5%). Cho et al. reported longer LT80 (100 h) by means of replacing only DDCzTrz with 2,3,4,5,6‐penta(9H‐carbazol‐9‐yl)benzonitrile.^11^ However, they still employed the same device structure using 3,3′‐di(9H‐carbazol‐9‐yl)‐1,1′‐biphenyl (mCBP),^10^ and its EQE was likely low. However, one cannot know that because they provided only lifespan of the OLED employing mCBP. On the other hand, all the presented EQE were achieved with the bis[2‐(diphenylphosphino)phenyl] ether oxide (DPEPO)‐host‐based OLEDs. Zhang et al. took one of the most meaningful steps forward commercialization of TADF OLEDs. They achieved high EQE_max_ and long device lifetime simultaneously with blue TADF emitters which are sterically shielded to reduce concentration quenching effect.^12^ To the best of our knowledge, they are the best blue TADF emitters in their specific colors so far. However, their EQEs at a practical brightness are significantly decreased compared to their EQE_max_. In spite of the very short delayed fluorescence lifetimes of the TADF emitters, their efficiency roll‐off is severe. It indicates their poor charge balance and it is mainly caused by their mismatched host, mCBP. We thus believe that there has been no report presenting practically long lifespan with blue TADF OLEDs simultaneously exhibiting sufficiently high EQE at a practical brightness yet although lifespan is as important as efficiency for practical success of OLED technology and it is due to lack of host materials well‐fitted to given TADF emitters for effective device engineering to achieve improved charge balance.

Here, we introduce an alternative host material to achieve a high‐efficiency and long‐lifespan blue TADF OLEDs at a relevant brightness as suggesting the appropriateness of replacing DPEPO with alternative host materials in development of blue TADF emitters.

Although we could suggest out various reasons for the scarce articles reporting the lifespan of blue TADF OLEDs, we here focus on the fact that there have been no good host materials well‐fitted for high‐*S*_1_, ‐*T*_1_ blue TADF emitters yet. In most papers, DPEPO has been employed mostly because it can boost TADF phenomenon resulting in high EQE_max_.^5^, ^9^, ^10^, ^11^, ^12^ Even in the papers reporting the 52 h and 100 h lifespan,^10^, ^11^ the high EQE_max_ OLEDs were achieved only with the DPEPO providing no device lifespan data while the long‐lifespan OLEDs were achieved with mCBP providing no efficiency data. Although DPEPO has been reported as an electron transport (ET)‐type host,^13^ DPEPO has a quite shallow lowest unoccupied molecular orbital (LUMO) and so all the TADF emitters doped in DPEPO have deeper LUMO than DPEPO. Both carriers cannot thus help being trapped in or transported through the emitters at least in part. It makes us lose one of the advantages of the host‐dopant system letting dopant molecules reduce electrical stress. In this paper, we present a long lifespan of a blue TADF OLED introducing an alternative host material. Simultaneously it exhibits practically high EQE which is much higher than those of fluorescent OLEDs. Its LT80 of 21 h was achieved with 8.7% EQE at the initial luminance (500 cd m^−2^) using 5,8‐bis(4‐(2,6‐diphenylpyrimidin‐4‐yl)phenyl)‐5,8‐dihydroindolo[2,3‐c]carbazole (BDpyInCz) (**Figure** **1**a)^14^ and 3′,5‐di(9H‐carbazol‐9‐yl)‐[1,1′‐biphenyl]‐3‐carbonitrile (mCBP‐CN) (Figure 1a)^15^ as an emitter and a host, respectively.

**Figure 1:**
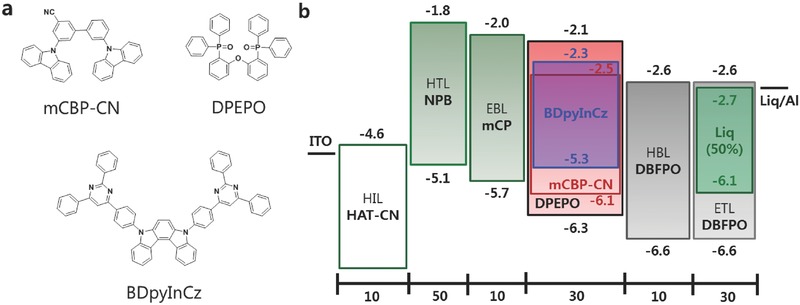
a) Chemical structures. b) Energy diagram of the device structures.

mCBP‐CN is well‐fitted for BDpyInCz having shallow LUMO and the highest occupied molecular orbital (HOMO) because mCBP‐CN has an ET‐type character with deep LUMO, and so the emitting layer composed of mCBP‐CN and the highly doped BDpyInCz works like a mixed host system. The electrons are mainly injected into and transported through the ET‐type host material while a considerable number of holes are probably injected into TADF emitters and transported through the percolation pathway composed of the emitters having a hole transporting (HT)‐type character in our device (Figure 1b). To achieve ET‐type host having deep LUMO, which is fitted for the HT‐type TADF emitters, we have tried to synthesize and attach cyano‐groups to various positions of mCBP. It is expected to have efficient electron‐injection and electron‐transport due to the strong electron‐withdrawing character of the cyano‐group and resultingly to improve OLED performances. They are also known to have good electrochemical and thermal stability.^16^

The mCBP‐CN‐based OLED exhibits a slight blue shift comparing to DPEPO‐based one while increasing the content of BDpyInCz induces a slight red‐shift in EL (**Figure** **2**a). The diode turn‐on voltages and OLED operating voltages of the mCBP‐CN‐based OLEDs at 500 cd m^−2^ are quite lower than those of the DPEPO‐based ones (Figure 2b). The smaller *E*_g_ and deeper LUMO of mCBP‐CN and the contribution of BDpyInCz to hole‐transport lead to the lower operating voltage of the mCBP‐CN‐based OLEDs. The DPEPO‐based OLEDs show severe 10–500 cd m^−2^ efficiency roll‐off of 75.6%, 52.5%, and 44.7% for 10%, 15%, and 20% doping ratio, respectively. Although EQE_max_ of the mCBP‐CN‐based device is lower than that of the DPEPO‐based one, its EQE at 500 cd m^−2^ is higher due to the better charge balance. Still severe efficiency roll‐off (40.8%, 38.5%, and 36.0% for 10%, 15%, and 20% doping ratio) of the mCBP‐CN‐based TADF OLEDs despite their improved charge balance is induced by severe triplet–triplet annihilation and singlet–triplet annihilation caused by the long excited‐state lifetime of BDpyInCz (τ_TADF_ = 32 µs).^17^ The efficiency roll‐off decreases according to the increased doping ratio without increment of driving voltage (*V*_d_) indicating charge trapping.^18^ This is additional evidence that BDpyInCz works like an HT‐type host forming a mixed host‐like system with both hosts. Detailed device characteristics are summarized in **Table** **1**. The role of BDpyInCz as a hole transporting pathway is verified again by significantly decreased *V*_d_ of the hole‐only devices (HODs) of mCBP‐CN:BDpyInCz and DPEPO:BDpyInCz compared to those of mCBP‐CN and DPEPO (Figure 2c).

**Figure 2:**
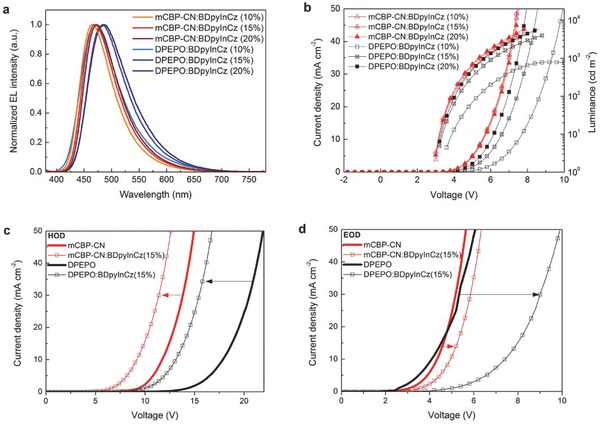
a) EL spectra of the DPEPO:BDpyInCz and mCBP‐CN:BDpyInCz devices with 10%, 15%, and 20% (by volume) content of BDpyInCz. b) Their current density–voltage and luminance–voltage curves. Current density–voltage curves of c) the HODs and d) the EODs of DPEPO, mCBP‐CN, DPEPO:BDpyInCz, and mCBP‐CN:BDpyInCz.

**Table 1:** Device performances of BDpyInCz‐based OLEDs

	*V*_d_ a) [V]	CIEx	CIEy	λ_EL_ b) [nm]	Quantum efficiency [%]			LT80 [h]
					10 cd m^−2^	100 cd m^−2^	500 cd m^−2^	
DPEPO:BDpyInCz (10%)	7.34	0.178	0.255	467	13.1	6.8	3.2	0.04
DPEPO:BDpyInCz (15%)	5.37	0.196	0.340	485	17.1	12.6	8.2	0.28
DPEPO:BDpyInCz (20%)	5.01	0.211	0.359	486	15.0	11.8	8.3	0.49
mCBP‐CN:BDpyInCz (10%)	4.95	0.162	0.204	464	12.0	9.5	7.1	8
mCBP‐CN:BDpyInCz (15%)	4.78	0.167	0.240	470	13.0	10.6	8.0	16
mCBP‐CN:BDpyInCz (20%)	4.74	0.173	0.266	472	13.6	11.2	8.7	21

^a)^ Driving voltage at 500 cd m^−2^

^b)^ EL wavelength.

In contrast with the HOD cases, *V*_d_ of the electron‐only devices (EODs) of mCBP‐CN:BDpyInCz and DPEPO:BDpyInCz are slightly higher than those of mCBP‐CN and DPEPO, respectively (Figure 2d). In the case of DPEPO, BDpyInCz works as a deep trap according to the significant increment of *V*_d_ (Figure 2d). However, in the case of mCBP‐CN, because LUMO of mCBP‐CN is deeper than that of BDpyInCz, we believe that the resistivity of the layer was possibly increased by the highly doped BDpyInCz (15%), decreasing the volume of mCBP‐CN and the main electron transporting pathway. The increased resistivity induced the slight increment of *V*_d_ (Figure 2d). The fact that the same phenomenon was observed in the EODs of mCBP‐2CN and mCBP‐3CN having deeper LUMO than that of mCBP‐CN supports our inference (Figure S20, Supporting Information). It is worthwhile to note that mCBP‐CN shows better transporting ability requiring lower *V*_d_ for the same current density for both hole and electron according to lower *V*_d_ of its HOD and EOD than those of DPEPO under the same current density (Figure 2c,d).

In addition to higher EQE at practical brightness, the mCBP‐CN:BDpyInCz device shows about 43 times longer lifespan than that of the DPEPO:BDpyInCz device (**Figure** **3**a). The longer lifespan was achieved due to its lower operating current at the same luminance and higher stability of mCBP‐CN than that of DPEPO. Figure 3b shows *V*_d_ of EODs under the same current density as a function of time for comparison of the solid state electrochemical stability while Figure 3c shows *V*_d_ of HODs. Increment of *V*_d_, which is equivalent to increment of resistivity induced by material degradation,^19^ of the DPEPO‐based devices is larger than those of the mCBP‐CN‐based ones within the same operating time. The voltage change of EOD of DPEPO is much dramatic than HOD. It indicates that electron transport through DPEPO is a harsher stress to DPEPO than hole transport even though DPEPO has been known as an ET‐type host, whereas the voltage changes of both EOD and HOD of mCBP‐CN are negligible. It reveals that the mCBP‐CN layer degrades much slower than the DPEPO layer under the same electrical stress as we expected. We believe that the improved lifespan is attributed to the high electrochemical stability and ET‐type character of mCBP‐CN, which is well‐fitted for HT‐type BDpyInCz forming a mixed host system‐like emitting layer. It can be also inferred from the improvement in current density–voltage and luminance–voltage characteristics as the content of BDpyInCz increases (Figure 2b).

**Figure 3:**
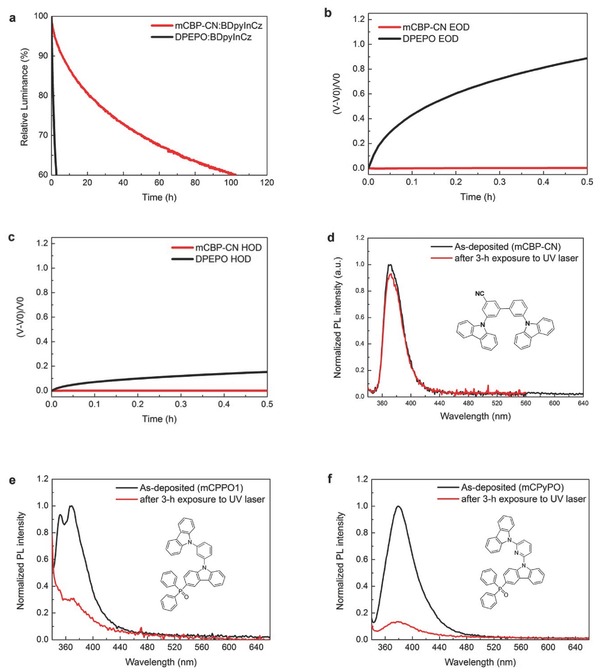
a) Luminance–time curves of the very DPEPO:BDpyInCz and mCBP‐CN:BDpyInCz devices, which exhibited EQE at 500 cd m^−2^ of 8.3% and 8.7%, respectively. Change of the driving voltages of b) EODs and c) HODs of DPEPO and mCBP‐CN keeping the driving current constant. *V*_0_ is the initial voltage. Comparison of PL spectra between as‐deposited and 3 h UV‐laser exposed films; d) mCBP‐CN, e) mCPPO1, and f) mCPyPO.

Because excited‐state unstability can be another source of OLED degradation,^20^ we performed photoluminescence (PL) stability study. Figure 3d shows normalized PL spectra of a 50 nm mCBP‐CN film on a quartz substrate measured after deposition and after 3 h UV‐laser‐exposure. The film was glass‐encapsulated in a nitrogen‐filled glove box right after the vacuum deposition. Normalization was performed by dividing them by the PL peak intensity of the as‐deposited film. The PL spectrum of the mCBP‐CN film after 3 h exposure to UV‐laser keeps its shape but loses peak intensity down to 0.93. On the other hand, we have unfortunately no result of the same experiment on DPEPO because we could not achieve PL from the DPEPO film because our highest‐energy UV‐laser (325 nm) cannot put the wide‐*E*_g_ DPEPO to excited state. Instead, we can presume that DPEPO is photochemically unstable by extrapolating from a report of low photochemical stability of phosphine oxide (PO) materials.^21^ In addition, we have a couple of phosphine oxide materials showing low PL stability such as (9‐(3‐(9H‐carbazol‐9‐yl)phenyl)‐9H‐carbazol‐3‐yl)diphenylphosphine oxide (mCPPO1)^22^ (Figure 3e) and (9‐(6‐(9H‐carbazol‐9‐yl)pyridin‐2‐yl)‐9H‐carbazol‐3‐yl)diphenylphosphine oxide (mCPyPO) (Figure 3f). They lose peak intensity down to 0.31 and 0.14, respectively. It indicates the PO materials are photochemically more unstable than mCBP‐CN in solid state. We expect that if we were able to introduce a certain TADF emitter exhibiting photochemically higher stability and shorter τ_TADF_ than those of BDpyInCz with mCBP‐CN, a longer device lifespan could be accomplished with higher EQE at a relevant brightness. The relative PL intensity after 3 h UV laser exposure and τ_TADF_ of BDpyInCz are 0.78 and 32 µs, respectively.

Although DPEPO lets blue TADF emitters exhibit their highest EQE_max_, it does not show any relevant performance at the practical brightness, nor even noticeable device lifespan except a report of Cui et al.^23^ Even in the paper, the devices employing DPEPO as a host have exhibited severer roll‐off and shorter device lifespan than those of another device employing a new host, bis(9,9′‐spirobifluorene‐2‐yl)ketone (SF3K) although they could remarkably improve device lifetime of TADF OLEDs based on DPEPO. In addition to the bubble in EQE_max_ of blue TADF OLEDs using DPEPO, which is less meaningful in the practical luminance range for display, we found out another weakness of the DPEPO‐based blue TADF study.

DPEPO produces a boost to TADF performance that is not replicated by hosts that are capable to enhance lifespan. For example, we present a BDpyInCz derivative, 12‐(4‐(2,6‐diphenylpyrimidin‐4‐yl)phenyl)‐5‐phenyl‐5,12‐dihydroindolo[3,2a] carbazole (DpyInCz). DpyInCz exhibits EQE_max_ of 8.8% with the same DPEPO‐based device structure as the BDpyInCz one (Figure 1b) as shown in **Figure** **4**a. However, it shows only 3.9% in EQE_max_ with mCBP‐CN‐based device structure. With such a low EQE_max_, we do not expect TADF working in the device. Furthermore, its efficiency roll‐off is much smaller than other devices (Figure 4a). Such a small efficiency roll‐off can be expected only in perfectly optimized phosphorescent OLEDs with a perfect charge balance in a mixed‐host system or traditional fluorescent OLEDs due to its extremely short excited‐state lifetime. It is not easy to achieve such a small efficiency roll‐off with phosphorescent and TADF emitters because of their long excited‐state lifetime.^17^, ^24^ The mCBP‐CN:DpyInCz device is probably a fluorescent OLED and we verified it through the PL study.

**Figure 4:**
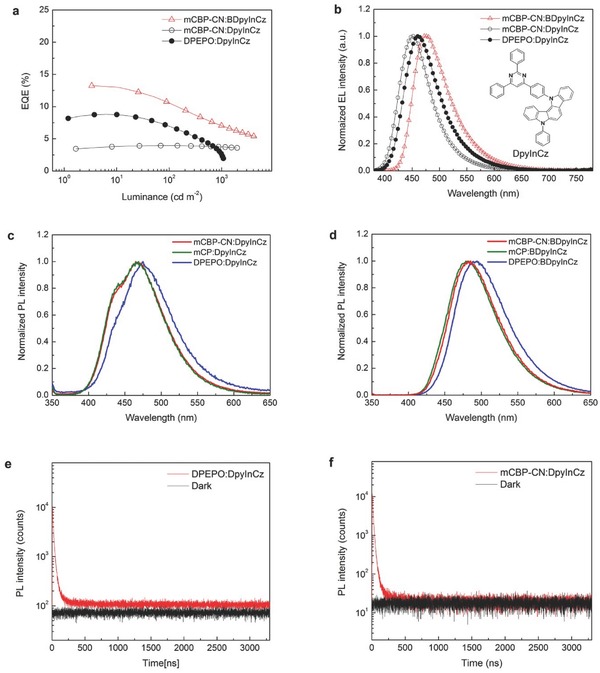
a) EQE‐luminance curves of the OLEDs based on mCBP‐CN:BDpyInCz, mCBP‐CN:DpyInCz, and DPEPO:DpyInCz and b) their EL spectra. PL spectra of the films; c) DpyInCz and d) BDpyInCz in various hosts, respectively. e) Transient PL of the DPEPO:DpyInCz and f) mCBP‐CN:DpyInCz films (red curves), respectively. Black curves named “Dark” were obtained during the same time period under the same measurement condition without irradiation.

While the EL spectra of DpyInCz‐based OLEDs show single peaks for both mCBP‐CN and DPEPO (Figure 4b), the PL spectra of mCBP‐CN:DpyInCz and DPEPO:DpyInCz films show shoulder peaks in shorter wavelength region (Figure 4c). Note that the shoulder of mCBP‐CN:DpyInCz film shows much higher intensity than that of DPEPO:DpyInCz. On the other hand, all the PL spectra of the BDpyInCz‐based films show single peaks (Figure 4d) analogous to the EL spectra of the BDpyInCz‐based OLEDs (Figure 2b). Interestingly, although DpyInCz exhibited delayed fluorescence in DPEPO (Figure 4e), we observed no meaningful delayed fluorescence from the mCBP‐CN:DpyInCz film (Figure 4f). Actually, no meaningful delayed fluorescence was observed from DpyInCz in all the host matrices we have tested, but in DPEPO. For instance, the 1,3‐di(9H‐carbazol‐9‐yl)benzene (mCP):DpyInCz film shows a strong shoulder PL peak at the same wavelength and no delayed fluorescence. All other transient PL spectra including BDpyInCz‐ and DpyInCz‐based films are provided in the Supporting Information. It is also worthwhile to note that the wavelength of the main EL peak of the mCBP‐CN:DpyInCz OLED is close to that of the shoulder PL peak of the mCBP‐CN:DpyInCz film while the EL peak wavelength of the DPEPO:DpyInCz OLED is close to the main PL peak of the DPEPO:DpyInCz film. It indicates that in the mCBP‐CN:DpyInCz OLED, the shoulder PL turns into the main EL via fluorescence while the main PL becomes the main EL via TADF in the DPEPO:DpyInCz OLED. We thus believe that the shoulder PL comes out of a locally excited (LE) state of DpyInCz rather than its intramolecular charge transfer (ICT) state. The most effective approach to reduce Δ*E*_ST_ and so to increase RISC is to employ a twisted intramolecular charge transfer (TICT) molecule having different dihedral angles between electron donating and accepting moieties leading to spatial separation of HOMO and LUMO phores.^6^, ^25^ The difference of dihedral angles can be changed by electrostatic interactions with host molecules. It is possible to produce various forms of chemical structures called rotamers, a class of conformers, in TICT molecules.^26^ Changing host may form various rotamers and exhibit dual fluorescence (Figure 4c). Lippert et al. discovered the dual fluorescence strongly depending on the solvent polarity^27^ and so we presume that the host matrix can also affect the phenomenon in the same way. We can find another evidence of the TICT transition in Figure 4d, noticeable change of the PL peak wavelength of the TADF emitters according to the employed hosts. The TADF emitters are working in the various hosts in the same way as in various solvents having different polarities. Even though we do not have any helpful information of the polarity of DPEPO, it seems safe to think DPEPO has an appropriate polarity to suppress dual fluorescence with LE transition and to manifest ICT transition of DpyInCz. In addition, according to more significant PL shift for DPEPO than other hosts (Figure 4d), we can presume that the polarity of DPEPO is quite different from those of other hosts. We will not discuss the polarity of DPEPO which may suppress dual fluorescence further because it is not our primary interest in this work. The important message is that certain emitters work only with DPEPO which is hardly employed for practical or commercial applications and it means DPEPO does not have an ability to screen out practically useless candidates for TADF emitters. We thus believe DPEPO must be replaced in the study of blue TADF OLEDs from now except for estimation of the maximum ability of TADF emitters in terms of EQE.

TICT molecules, the most popular TADF emitters, have a potential risk that some of them possibly work only with DPEPO or DPEPO‐like host materials suppressing dual fluorescence. This bias in selecting a host material leaves no room for utilizing device engineering to educe the potential ability of a given TADF emitter. Because of the limitation, we cannot say the newly reported DpyInCz in this work is practically useful though DpyInCz works anyhow in the DPEPO matrix as a blue TADF emitter. We would thus like to suggest that it is better to replace DPEPO with a stable host well‐fitted for a given blue TADF emitter to accelerate the advance of TADF technology. The introduced mCBP‐CN successfully replaces DPEPO and helps accomplish a remarkably long lifespan in a blue TADF OLED with high efficiency at a practical brightness as educing the potential ability of a given blue TADF emitter despite its relatively long τ_TADF_.

## Supporting information

Supplementary
