# Supplementary material for: An Alternative Host Material for Long‐Lifespan Blue Organic Light‐Emitting Diodes Using Thermally Activated Delayed Fluorescence
Source: Adv Sci (Weinh). 2017 Mar 23;4(8):1600502. doi: 10.1002/advs.201600502 (PMC5566347; doi:10.1002/advs.201600502)
Supplement: Supplementary file 1 — Supplementary [file ADVS-4-na-s001.pdf]

Copyright WILEY-VCH Verlag GmbH & Co. KGaA, 69469 Weinheim, Germany, 2017.

ADVANCED SCIENCE
Open Access

Supporting Information

for *Adv. Sci.,* DOI: 10.1002/advs.201600502

An Alternative Host Material for Long-Lifespan Blue Organic Light-Emitting Diodes Using Thermally Activated Delayed Fluorescence

Soo-Ghang Ihn, Namheon Lee, Soon Ok Jeon, Myungsun Sim, Hosuk Kang, Yongsik Jung, Dal Ho Huh, Young Mok Son, Sae Youn Lee, Masaki Numata, Hiroshi Miyazaki, Rafael Gómez-Bombarelli, Jorge Aguilera-Iparraguirre, Timothy Hirzel, Alán Aspuru-Guzik, Sunghan Kim, and Sangyoon Lee**

Copyright WILEY-VCH Verlag GmbH & Co. KGaA, 69469 Weinheim, Germany, 2016.

Supporting Information

An alternative host material for long-lifespan blue organic light-emitting diodes using thermally activated delayed fluorescence

Soo-Ghang Ihn, Namheon Lee, Soon Ok Jeon, Myungsun Sim, Hosuk Kang, Yongsik Jung, Dal Ho Huh, Young Mok Son, Sae Youn Lee, Masaki Numata, Hiroshi Miyazaki, Rafael Gómez-Bombarelli, Jorge Aguilera- Iparraguirre, Timothy Hirzel, Alán Aspuru-Guzik, Sunghan Kim, and Sangyoon Lee**

1 Material synthesis

1.1 General procedures

Chemicals were purchased from Sigma-Aldrich Co., Tokyo Chemical Industry Co., and Wako Pure Chemical Industries Ltd., and used without further purification. ^{1}H NMR and ^{13}C NMR spectra were recorded on a Bruker ASCEND 500 at 500 MHz using CD_2Cl_2 as the solvent. The MLADI-TOF mass spectra were recorded using a Bruker Ultraflex III TOF/TOF 200 spectrometer.

1.2 Synthesis of BDPyInCz

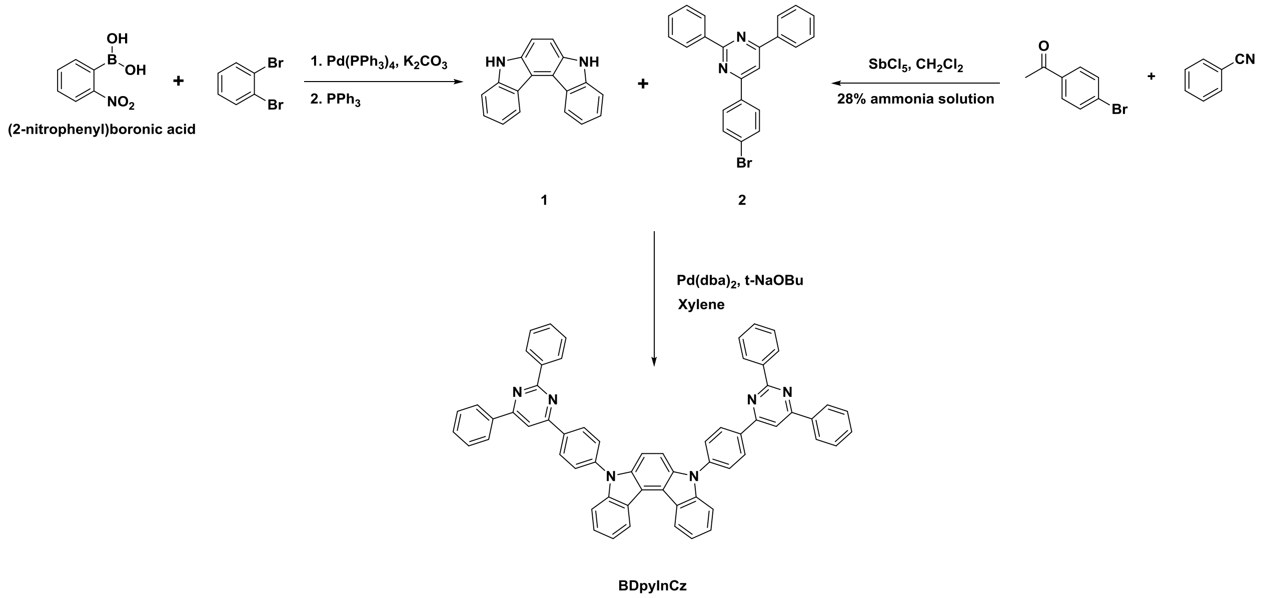

Scheme 1. Synthetic route of **BDPyInCz**

1. 5,8-Dihydroindolo[2,3-c]carbazole

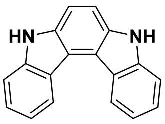

A mixture of (2-nitrophenyl)boronic acid (3.6 g, 21.94 mmol), 1,2-dibromobenzene (2.3 g, 9.75 mmol) and tetrakis(triphenylphosphine)palladium(0) (0.56 g, 5 mol%) in 25 mL of anhydrous toluene was refluxed under argon for 24 h. To the reaction mixture was slowly added a solution of potassium carbonate (4.0 g, 29.25 mmol) in 25 mL of water. After allowing it to cool to ambient temperature, the reaction mixture was extracted with chloroform and water. The organic layer was evaporated with a rotary evaporator. The product was purified by column chromatography using chloroform/*n*-hexane (1/1) and 1.5 g of crude product and triphenylphosphine (5.0 g, 19.50) in 30 mL of dichlorobenzene was heated 160 ^{o}C under argon for 12 h. After completion of the reaction, the reaction mixture was diluted with MeOH (200 mL) and filtered. The product was purified by column chromatography using chloroform/*n*-hexane (1/3) and 0.8 g (31%) of 5,8-dihydroindolo[2,3-c]carbazole (**1**) was obtained a white solid.

2. 4-(4-Bromophenyl)-2,6-diphenylpyrimidine

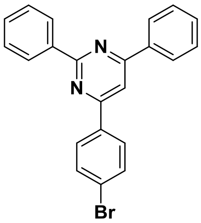

A mixture of 4-bromoacetophenone (3.5 g, 17.58 mmol) and benzonitrile (1.83 g, 17.58 mmol) in dichloromethane (40 mL) cooled at 0 ^{o}C by ice bath and stirred for 30 min. Then, antimony(V) chloride (10.51 g, 35.17 mmol) was added dropwise to the above solution. The mixture was stirred at room temperature for 1 h and further stirred and refluxed overnight. The cooled mixture was filtrated, and the collected yellow solid was washed by dichloromethane. The solid was slowly added to 75 mL of 28% ammonia solution cooled at 0

^{o}C by ice bath and stirred for 30 min. Then the mixture was stirred for 3h at room temperature. Subsequently, the mixture was filtrated, and the collected white solid was washed by water. Then the solvent was removed under vacuum, and 6.5 g (95%) of 4-(4-bromophenyl)-2,6-diphenylpyrimidine (**2**) was obtained as a white solid.

5,8-Bis(4-(2,6-diphenylpyrimidin-4-yl)phenyl)-5,8-dihydroindolo[2,3-c]carbazole (BDPyInCz)

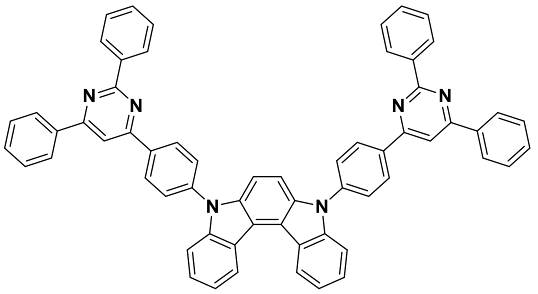

A mixture of 5,8-dihydroindolo[2,3-c]carbazole (**1**) (5.0 g, 19.51 mmol), 4-(4-Bromophenyl)-2,6-diphenylpyrimidine (**2**) (16.62 g, 42.92 mmol), and sodium-*tert*-butoxide (3.75 g, 39.02 mmol) in xylene (50 mL) was added and stirred at 165 ^{o}C under nitrogen for 12 h. To the reaction mixture was slowly added a solution of $Pd(dba)_2$ (1.12 g, 1.95 mmol), and tri-*tert*-butylphosphine (0.789 g, 3.9 mmol) in 15 mL of xylene. After allowing it to cool to room temperature, the reaction mixture was diluted with MeOH (200 mL) and filtered. The reaction mixture was carefully washed with water/MeOH (100/100 mL). The resulting brown solid was collected by filtration. The crude product was purified by column chromatography using dichloromethane/*n*-hexane (1/3) as eluent. The yellow solid was obtained then the product was recrystallized from toluene and finally dried under vacuum to give 5,8-bis(4-(2,6-diphenylpyrimidin-4-yl)phenyl)-5,8-dihydroindolo[2,3-c]carbazole (**BDPyInCz**) as a yellow crystal in 11.9 g (70%) yield. ^{1}H NMR (500 MHz, CD_2Cl_2): δ (ppm) 9.022−9.006 (d, *J* = 8.0Hz, 2H), 8.784−8.768 (d, *J* = 8.0Hz, 4H), 8.636−8.619 (d, *J* = 8.5Hz, 4H), 8.380−8.368 (d, *J* = 6.0Hz, 4H), 8.202 (s, 2H), 7.901−7.884 (d, *J* = 8.5Hz, 4H), 7.685−7.512 (m, 8H), 7.618-7.517 (m, 12H); ^{13}C NMR (500 MHz, CD_2Cl_2): δ (ppm) 165.6, 165.1, 164.4, 141.4, 140.9,

138.7, 138.0 137.2, 131.5, 131.4 129.5, 129.1 129.0 128.6, 127.9, 126.1, 124.1, 123.7, 120.5, 117.7, 111.0, 110.6, 109.7; MALDI-TOF/MS: 870 [(M + H)$^+$].

1.3 Synthesis of DPyInCz

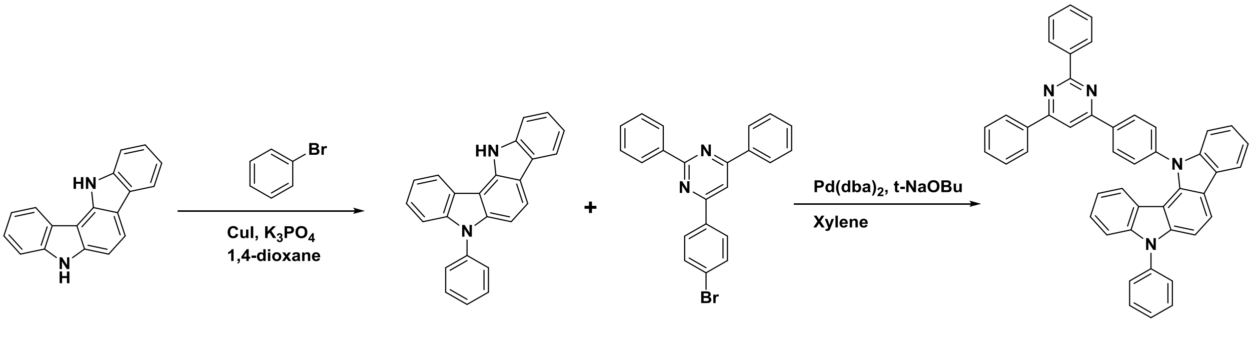

3 2 DpyInCz

Scheme 2. Synthetic route of **DPyInCz**

3. 5-Phenyl-5,12-dihydroindolo[3,2-a]carbazole

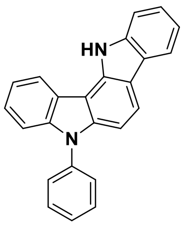

A mixture of 5,12-dihydroindolo[3,2-a]carbazole (15 g, 58.52 mmol), bromobenzene (9.65 g, 61.45 mmol), CuI (0.557 g, 2.93 mmol), trans-1,2-diaminocyclohexane (1.337 g, 11.70 mmol), and potassium phosphate (18.63 g, 87.78 mmol) in dioxane (300 mL) was stirred at 110 ^{o}C for 12 h. After allowing it to cool to room temperature, the reaction mixture was diluted with toluene (500 mL) and filtered with celite. The reaction mixture was evaporated and the crude product was purified by column chromatography on silica gel using dichloromethane/*n*-hexane (1/9) as eluent. The yellow solid obtained after evaporating the solvent was recrystallized from toluene and finally dried under vacuum to give 5-phenyl-5,12-dihydroindolo[3,2-a]carbazole as a white powder in 18.1 g (96%) yield.

12-(4-(2,6-Diphenylpyrimidin-4-yl)phenyl)-5-phenyl-5,12-dihydroindolo[3,2-a]carbazole (DpyInCz)

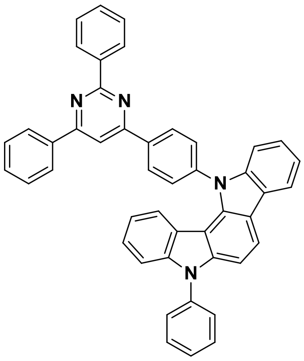

12-(4-(2,6-Diphenylpyrimidin-4-yl)phenyl)-5-phenyl-5,12-dihydroindolo[3,2-a]carbazole (**DpyInCz**) was obtained as yellow crystal (19.29 g, 78%) from 4-(4-bromophenyl)-2,6-diphenylpyrimidine (**2**) (15 g, 38.73 mmol) and 5-phenyl-5,12-dihydroindolo[3,2-a]carbazole (**3**) (14.16 g, 42.60 mmol) using a procedure analogous to that used for 5,8-bis(4-(2,6-diphenylpyrimidin-4-yl)phenyl)-5,8-dihydroindolo[2,3-c]carbazole (**BDPyInCz**). ^{1}H NMR (500 MHz, CD_2Cl_2): δ (ppm) 8.806−8.787 (dd, *J* = 7.0Hz, 2.0Hz, 2H), 8.670−8.653 (dd, *J* = 8.5Hz, 2.0Hz, 2H), 8.396−8.377 (dd, *J* = 7.5Hz, 2.0Hz, 2H), 8.245−8.168 (m, 3H), 7.872−7.855 (dd, *J* = 8.5Hz, 2.0Hz, 2H), 7.689−7.668 (td, *J* = 7.0Hz, 2.0Hz, 2H), 7.639−7.551 (m, 9H), 7.505−7.490 (d, *J* = 7.5Hz, 1H), 7.422−7.346 (m, 3H), 7.137−7.301 (d, *J* = 8.0Hz, 1H), 7.207−7.189 (td, *J* = 8.0Hz, 1.0Hz, 1H), 6.800−6.768 (td, *J* = 7.0Hz, 1.0Hz, 1H), 6.282−6.266 (d, *J* = 7.0Hz, 1H); ^{13}C NMR (500 MHz, CD_2Cl_2): δ (ppm) 165.60, 165.10, 164.26, 143.22, 142.32, 141.15, 138.68, 138.19, 137.96, 137.76, 137.10, 131.52, 131.33, 130.55, 129.69, 129.52, 129.32, 129.08, 128.99, 128.60, 128.55, 127.87, 125.32, 125.05, 125.01, 124.09, 121.76, 121.34, 119.95, 119.52, 118.97, 118.27, 110.96, 110.76, 109.85, 108.44, 104.59; MALDI-TOF/MS: 639 [$(M + H)^+$].

1.4 Synthesis of mCBP-CN, mCBP-2CN and mCBP-3CN

4. ***3-Bromo-5-(9H-carbazol-9-yl)benzonitrile***

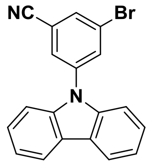

A solution of 9H-carbazole (5 g, 29.90 mmol) in anhydrous DMF was dropwised into dispersion of sodium hydride (60%, 0.718 g, 29.90 mmol) in anhydrous DMF in ice bath. After stirring for 1 h, 3-bromo-5-fluorobenzonitrile (6.55 g, 32.89 mmol) was dissolved in anhydrous DMF (150 mL) and it was added to the stirred reaction mixture under a nitrogen atmosphere. The solution was allowed to be stirred at 120 ^{o}C for 12 h. The reaction mixture was extracted with dichloromethane and distilled water, and then the separated dichloromethane layer was dried using anhydrous magnesium sulfate and concentrated in vacuo. The reaction product was purified by silica gel chromatography using a mixture of chloroform and *n*-hexane (1/2) as an eluent. A white product was obtained in 8.8 g (85%) yield.

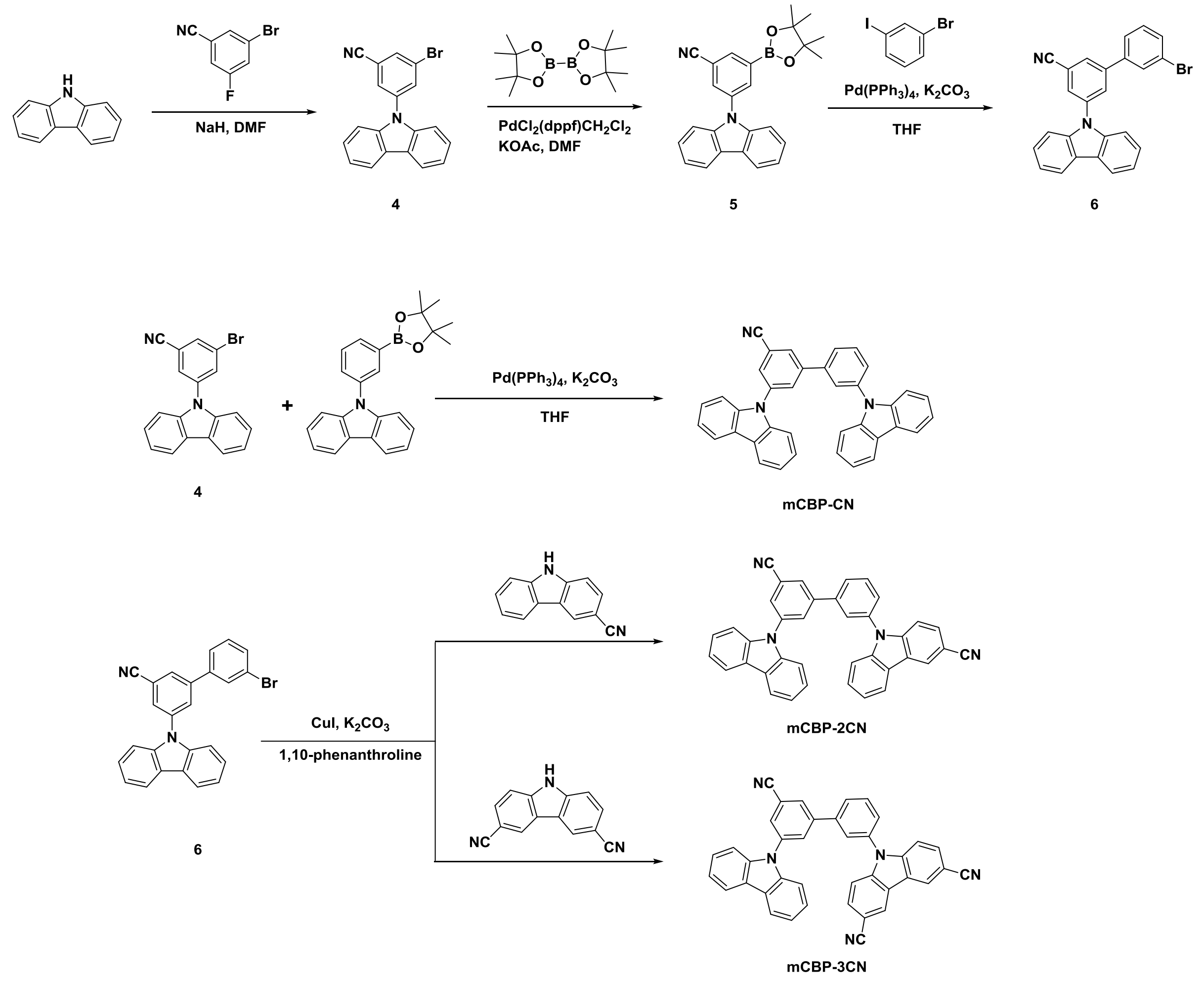

Scheme 3. Synthetic route for **mCBP- CN**, **mCBP-2CN** and **mCBP-3CN**

5. ***3-(9H-Carbazol-9-yl)-5-(4,4,5,5-tetramethyl-1,3,2-dioxaborolan-2-yl)benzonitrile***

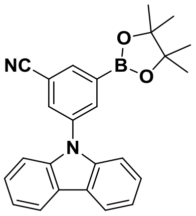

3-Bromo-5-(9H-carbazol-9-yl)benzonitrile (8.00g, 23.04 mmol), bis(pinacolato)diboron (7.02 g, 27.65 mmol), potassium acetate (6.78 g, 69.21 mmol), and $PdCl_2(dppf)CH_2Cl_2$ (0.84 g, 1.15 mmol) were dissolved in DMF (110 mL) under nitrogen atmosphere. The reaction mixture was stirred and refluxed for 12 h. The mixture was filtered, diluted with ethyl acetate, and washed with water. The organic layer was dried over anhydrous $MgSO_4$ and evaporated with a rotary evaporator. The crude product was purified by column chromatography by

dichloromethane/*n*-hexane (1/1) and the dried under vacuum to give 3-(9H-carbazol-9-yl)-5-(4,4,5,5-tetramethyl-1,3,2-dioxaborolan-2-yl)benzonitrile (**5**) (7.3 g, 80%).

6. ***3'-Bromo-5-(9H-carbazol-9-yl)-[1,1'-biphenyl]-3-carbonitrile***

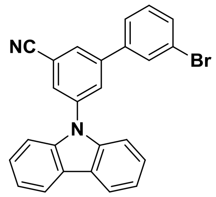

A mixture of 1-bromo-3-iodobenzene (4 g, 14.14 mmol), 3-(9H-carbazol-9-yl)-5-(4,4,5,5-tetramethyl-1,3,2-dioxaborolan-2-yl)benzonitrile (**5**) (6.69 g, 16.97 mmol) and tetrakis(triphenylphosphine)palladium(0) (0.82 g, 5 mol%) in 35 mL of anhydrous tetrahydrofuran was refluxed under nitrogen for 12 h. To the reaction mixture was slowly added a solution of potassium carbonate (5.86 g, 42.42 mmol) in 35 mL of water. After allowing it to cool to ambient temperature, the reaction mixture was diluted with MeOH (100 mL) and filtered and dried. The crude product was purified by column chromatography using dichloromethane/*n*-hexane (2/1) and 4.7 g (78%) of 3'-bromo-5-(9H-carbazol-9-yl)-[1,1'-biphenyl]-3-carbonitrile (**6**) was obtained a white solid.

3',5-Di(9H-carbazol-9-yl)-[1,1'-biphenyl]-3-carbonitrile (mCBP-CN)

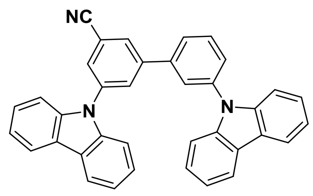

3',5-di(9H-carbazol-9-yl)-[1,1'-biphenyl]-3-carbonitrile (**mCBP-CN**) was obtained as white crystal (5.57 g, 73 %) from 3-bromo-5-(9H-carbazol-9-yl)benzonitrile (**4**) (5.2 g, 14.98 mmol) and 9-(3-(4,4,5,5-tetramethyl-1,3,2-dioxaborolan-2-yl)phenyl)-9H-carbazole (6.64 g, 17.97 mmol) using a procedure analogous to that used for 3'-bromo-5-(9H-carbazol-9-yl)-[1,1'-biphenyl]-3-carbonitrile (**6**). ^{1}H NMR (500 MHz, CD_2Cl_2): δ (ppm) 8.173-8.146 (m, 5H), 8.043 (s, 1H), 7.928 (s, 1H), 7.883 (s, 1H), 7.782-7.769 (m, 2H), 7.697-7.684 (m, 1H),

7.485−7.413 (m, 8H), 7.350−7.291 (m, 4H); ^{13}C NMR (500 MHz, CD_2Cl_2): δ (ppm) 144.17, 141.33, 140.91, 140.65, 140.03, 139.32, 131.50, 130.62, 130.02, 129.83, 127.98, 126.95, 126.75, 126.69, 126.24, 124.30, 124.01, 121.34, 121.05, 120.89, 120.76,118.41, 115.42, 110.18, 109.97; MALDI-TOF/MS: 510 [(M + H)$^+$].

9-(3'-(9H-Carbazol-9-yl)-5'-cyano-[1,1'-biphenyl]-3-yl)-9H-carbazole-3-carbonitrile (mCBP-2CN)

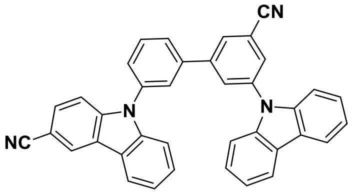

A mixture of 9H-carbazole-3-carbonitrile (2.17 g, 11.31 mmol), 3'-bromo-5-(9H-carbazol-9-yl)-[1,1'-biphenyl]-3-carbonitrile (**6**) (4.00 g, 9.43 mmol), CuI (0.90 g, 4.71 mmol), 1,10-phenanthroline (1.69 g, 9.43 mmol), and potassium carbonate (2.61 g, 18.85 mmol) in DMF (50 mL) was stirred at 160 ^{o}C for 12 h. After allowing it to cool to room temperature, the reaction mixture was diluted with MeOH (150 mL) and filtered and dried. The reaction mixture was evaporated and the crude product was purified by column chromatography on silica gel using dichloromethane/*n*-hexane (1/9) and 3.9 g (77%) of 9-(3'-(9H-carbazol-9-yl)-5'-cyano-[1,1'-biphenyl]-3-yl)-9H-carbazole-3-carbonitrile (mCBP-2CN) was obtained a white solid. ^{1}H NMR (500 MHz, CD_2Cl_2): δ (ppm) 8.481 (s, 1H), 8.200−8.181 (dd, *J* = 7.5Hz, 1.0Hz, 1H), 8.175−8.156 (dd, *J* = 7.5Hz, 1.0Hz, 2H), 8.438 (s, 1H), 8.038 (s, 1H), 7.936 (s, 1H), 7.844−7.791 (m, 3H), 7.685−7.643 (m, 2H), 7.539−7.430 (m, 7H), 7.411−7.379 (td, *J* = 7.0Hz, 1.0Hz, 1H), 7.354−7.322 (td, *J* = 7.0Hz, 1.5Hz, 2H); ^{13}C NMR (500 MHz, CD_2Cl_2): δ (ppm) 143.823, 143.072, 142.161, 140.960, 140.879, 140.081, 138.121, 131.776, 130.605, 130.001, 129.905, 128.106, 128.056, 127.716, 126.942, 126.344, 125.874, 124.301, 124.179, 122.884, 121.979, 121.382, 121.289, 121.072, 120.653, 118.327, 115.497, 111.018, 110.794, 109.908, 103.539; MALDI-TOF/MS: 535 [(M + H)$^+$].

9-(3'-(9H-Carbazol-9-yl)-5'-cyano-[1,1'-biphenyl]-3-yl)-9H-carbazole-3,6-dicarbonitrile (mCBP-3CN)

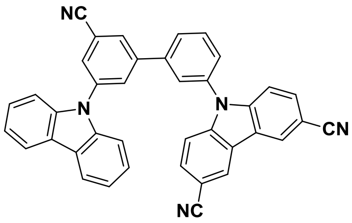

9-(3'-(9H-carbazol-9-yl)-5'-cyano-[1,1'-biphenyl]-3-yl)-9H-carbazole-3,6-dicarbonitrile (**mCBP-3CN**) was obtained as white crystal (2.54 g, 48%) from 3'-bromo-5-(9H-carbazol-9-yl)-[1,1'-biphenyl]-3-carbonitrile (**6**) (4.00 g, 9.43 mmol) and 9H-carbazole-3,6-dicarbonitrile (2.46 g, 11.31 mmol) using a procedure analogous to that used for 9-(3'-(9H-carbazol-9-yl)-5'-cyano-[1,1'-biphenyl]-3-yl)-9H-carbazole-3-carbonitrile (**mCBP-2CN**). ^{1}H NMR (500 MHz, CD_2Cl_2): δ (ppm) 8.516 (s, 2H), 8.175−8.159 (d, *J* = 8.0Hz, 2H), 8.124 (s, 1H), 8.031 (s, 1H), 7.957 (s, 1H), 7.893-7.878 (d, *J* = 7.5Hz, 1H), 7.855-7.823 (t, 8.5Hz, 1.5Hz, 1H), 7.798 (s, 1H), 7.773−7.753 (dd, *J*= 1.5Hz, 8.5Hz, 2H), 7.638−7.622 (d, *J* = 8.0Hz, 1H), 7.511−7.494 (d, *J* = 8.5Hz, 2H), 7.7456-7.444 (m, 4H), 7.354-7.338 (td, *J*= 6Hz, 2Hz , 2H); ^{13}C NMR (500 MHz, CD_2Cl_2): δ (ppm) 143.91, 143.54, 141.30, 140.89, 140.17, 137.06, 132.08, 131.24, 130.64, 130.21, 130.02, 128.59, 128.11, 126.95, 126.41, 126.33, 124.33, 123.11, 121.44, 121.11, 120.03, 118.27, 115.60, 111.80, 109.87, 105.16; MALDI-TOF/MS: 560 [$(M + H)^{+}$].

1.5 Synthesis of mCPyPO

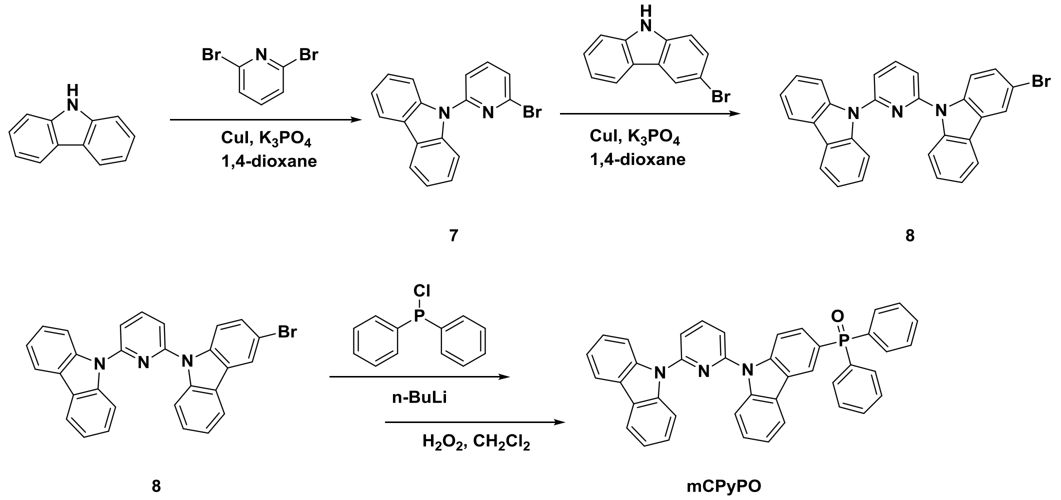

Scheme 4. Synthetic route of **mCPyPO**

7. 9-(6-Bromopyridin-2-yl)-9H-carbazole

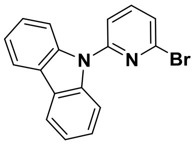

9-(6-Bromopyridin-2-yl)-9H-carbazole (**7**) was obtained as white powder (7.32 g, 54%) from 4-(4-bromophenyl)-2,6-dibromopyridine (11.91 g, 50.28 mmol) and 9H-carbazole (7 g, 41.90 mmol) using a procedure analogous to that used for 5-phenyl-5,12-dihydroindolo[3,2-a]carbazole (**3**).

8. 9-(6-(9H-carbazol-9-yl)pyridin-2-yl)-3-bromo-9H-carbazole

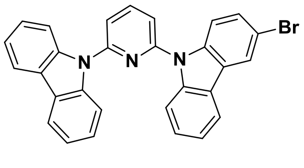

9-(6-(9H-carbazol-9-yl)pyridin-2-yl)-3-bromo-9H-carbazole (**8**) was obtained as white powder (4.2 g, 52%) from 9-(6-Bromopyridin-2-yl)-9H-carbazole (**7**) (5.3 g, 16.40 mmol) and 3-bromo-9H-carbazole (4.84 g, 19.68 mmol) using a procedure analogous to that used for 5-phenyl-5,12-dihydroindolo[3,2-a]carbazole (**3**).

(9-(6-(9H-carbazol-9-yl)pyridin-2-yl)-9H-carbazol-3-yl)diphenylphosphine oxide (mCPyPO)

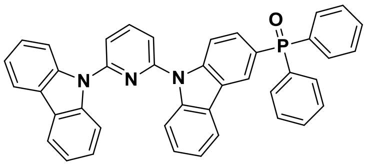

Into a 100 mL, two-neck flask, was placed a 9-(6-(9H-carbazol-9-yl)pyridin-2-yl)-3-bromo-9H-carbazole (**8**) (4.00 g, 8.19 mmol) in THF (40 mL). The reaction flask was cooled to -78°C and *n*-BuLi (2.5M in hexane, 4.26 mL) was added dropwise slowly. The whole solution was stirred at this temperature for 3 h, followed by addition of a solution of chlorodiphenylphophine (2.34 g, 10.65 mmol) under argon atmosphere. The resulting mixture was gradually warmed to ambient temperature and quenched by methanol (80 mL). The mixture was extracted with dichloromethane. The combined organic layers were dried magnesium sulfate, filtered, and evaporated under reduced pressure. The white powdery product was obtained to 3.42 g. A total of 9-(6-(9H-carbazol-9-yl)pyridin-2-yl)-3-(diphenylphosphanyl)-9H-carbazole, dichloromethane (20 mL), and hydrogen peroxide (5 mL) were stirred overnight at room temperature. The organic layer was separated and washed with dichloromethane and water. The extract was evaporated to dryness affording a white solid. ^{1}H NMR (500 MHz, CD_2Cl_2): δ (ppm) 8.566−8.539 (dd, *J* = 7.0Hz, 1.0Hz, 1H), 8.231−8.200 (t, 7.5Hz, 1H), 8.159−8.138 (m, 3H), 8.047−8.025 (dd, *J* = 7.0Hz, 1.5Hz, 1H), 7.985−7.957 (m, 3H), 7.737−7.694 (m, 5H), 7.671−7.635 (d, 7.5Hz, 1H), 7.627−7.545 (m, 3H), 7.501−7.464 (m, 5H), 7.437−7.403 (dd, *J* = 7.0Hz, 1.5Hz, 2H), 7.383−7.325 (m, 3H); ^{13}C NMR (500 MHz, CD_2Cl_2): δ (ppm)152.20, 151.29, 142.12, 124.10, 141.63, 140.64, 140.02, 134,55, 133.72, 132.60, 132.53, 132.32, 132.30, 130.16, 130.06, 129.05, 128.60, 127.65, 126.89, 125.44, 125.35, 125.02, 124.98, 124.91, 124.49, 124.34, 122.36, 121.84, 121.14, 120.70, 116.89, 116.50, 112.33, 112.21; MALDI-TOF/MS: 609 [(M + H)$^{+}$].

1.6 ^{1}H and ^{13}C NMR spectra of materials

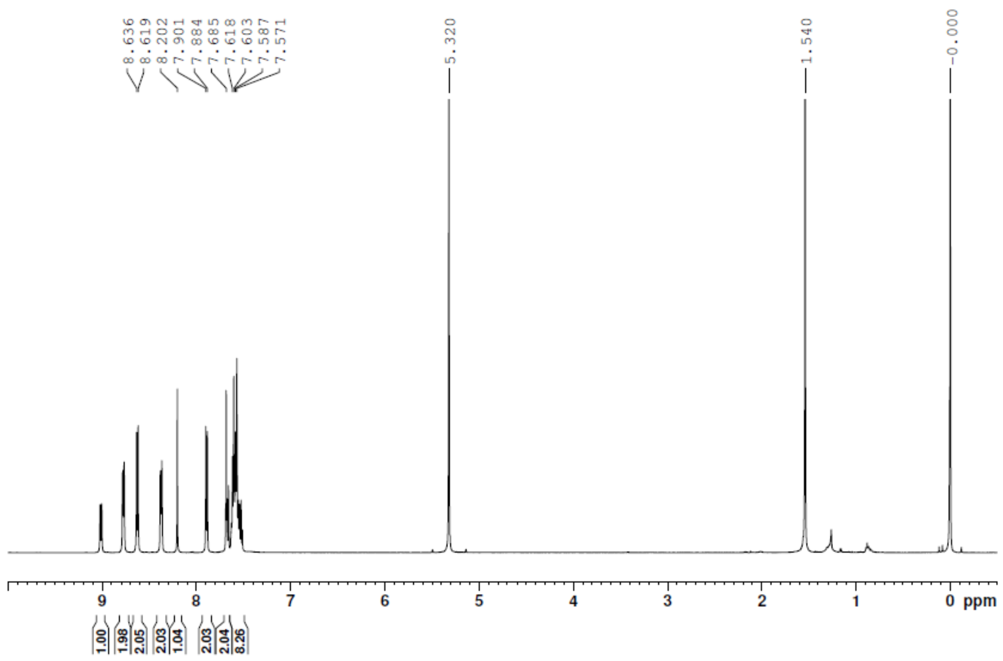

Figure S1. ^{1}H NMR spectra of **BDPyInCz**

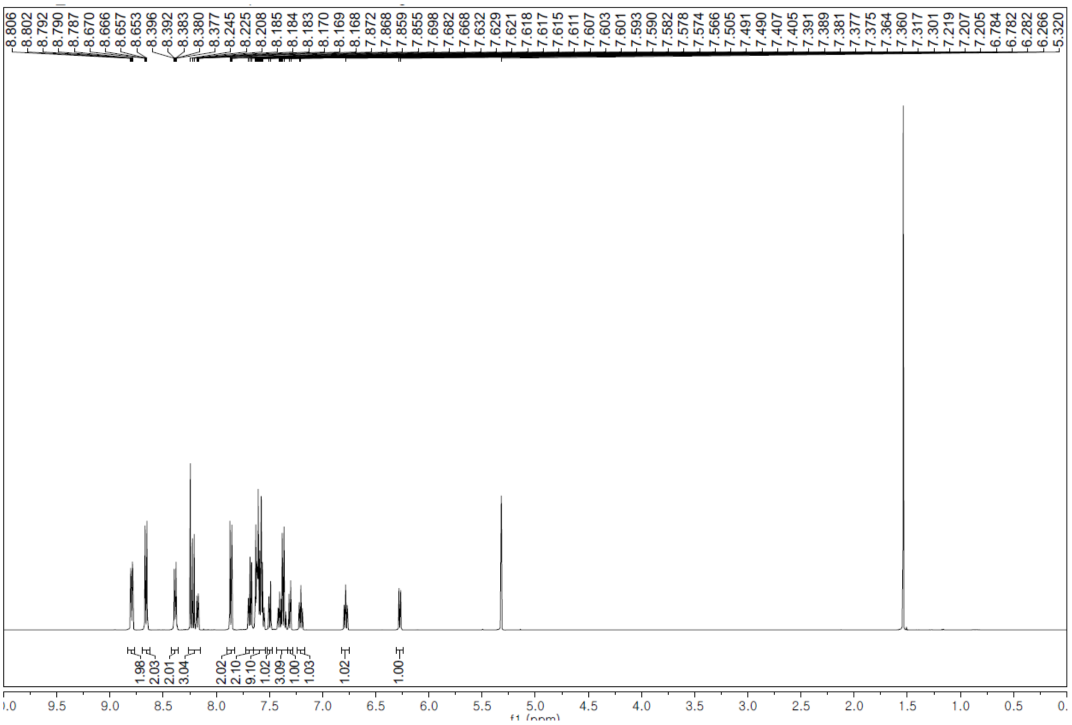

Figure S2. ^{1}H NMR spectra of **DpyInCz**

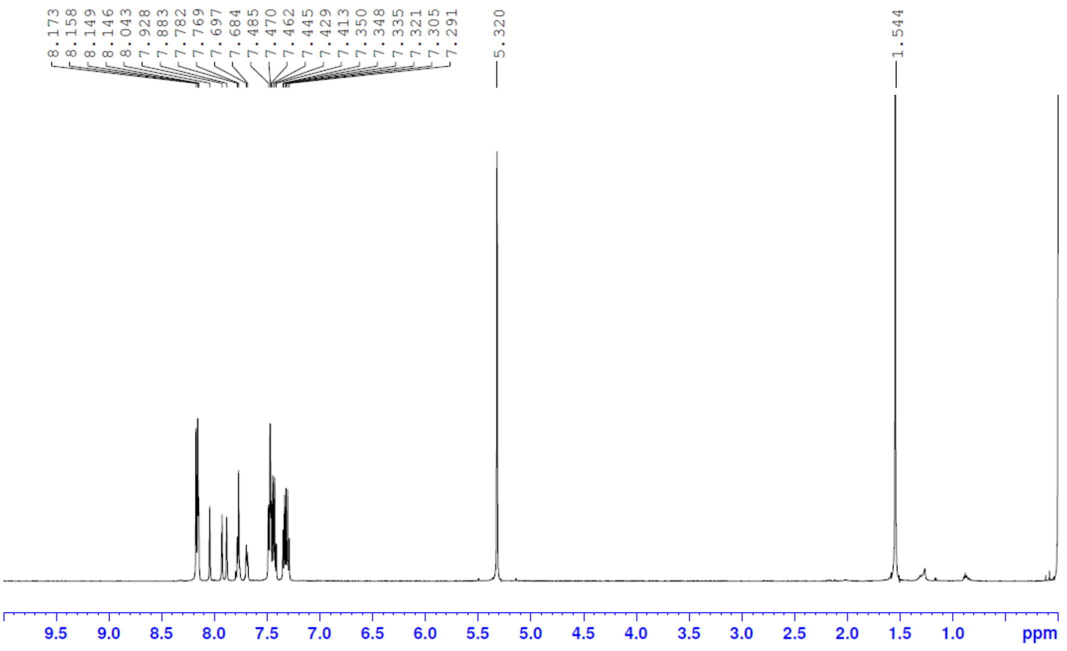

Figure S3. ^{1}H NMR spectra of **mCBP-CN**

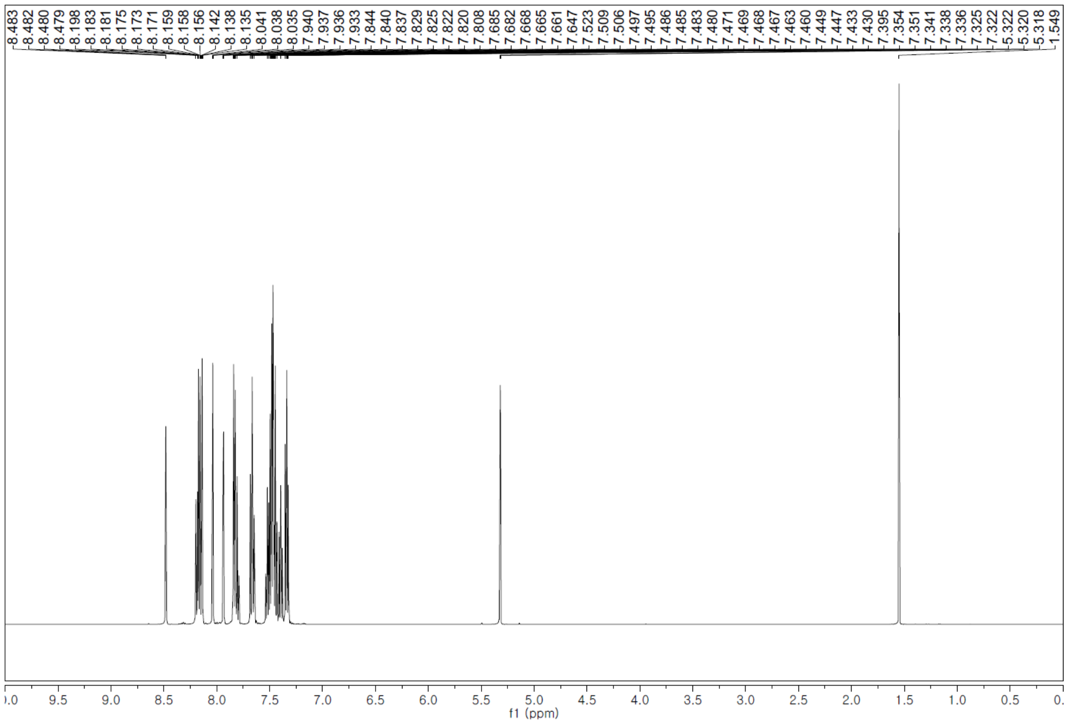

Figure S4. ^{1}H NMR spectra of **mCBP-2CN**

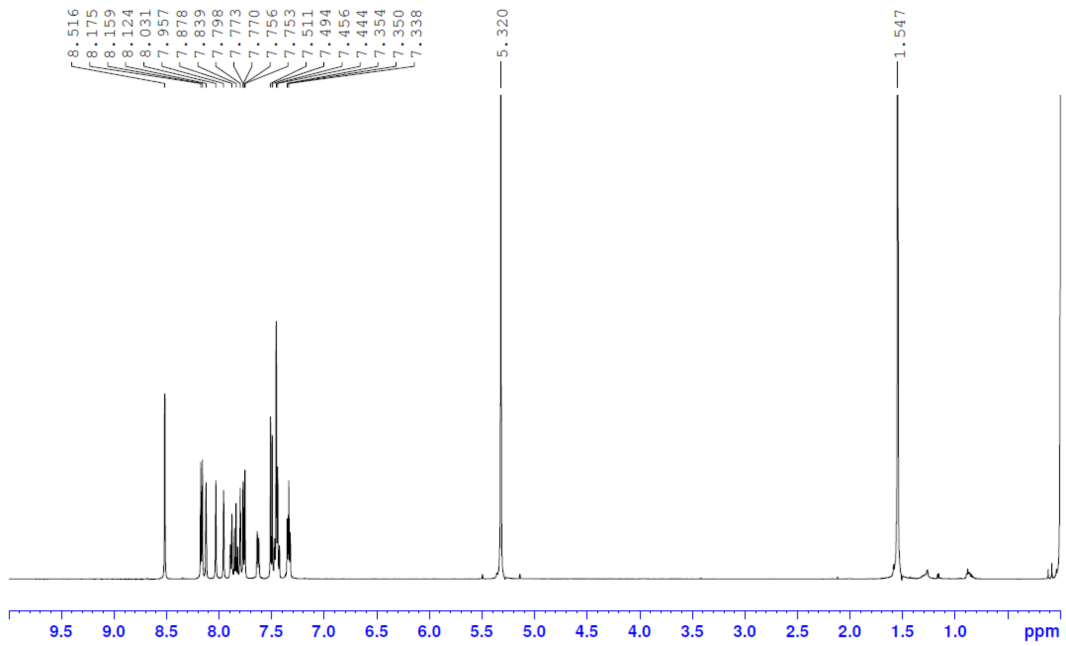

Figure S5. ^{1}H NMR spectra of **mCBP-3CN**

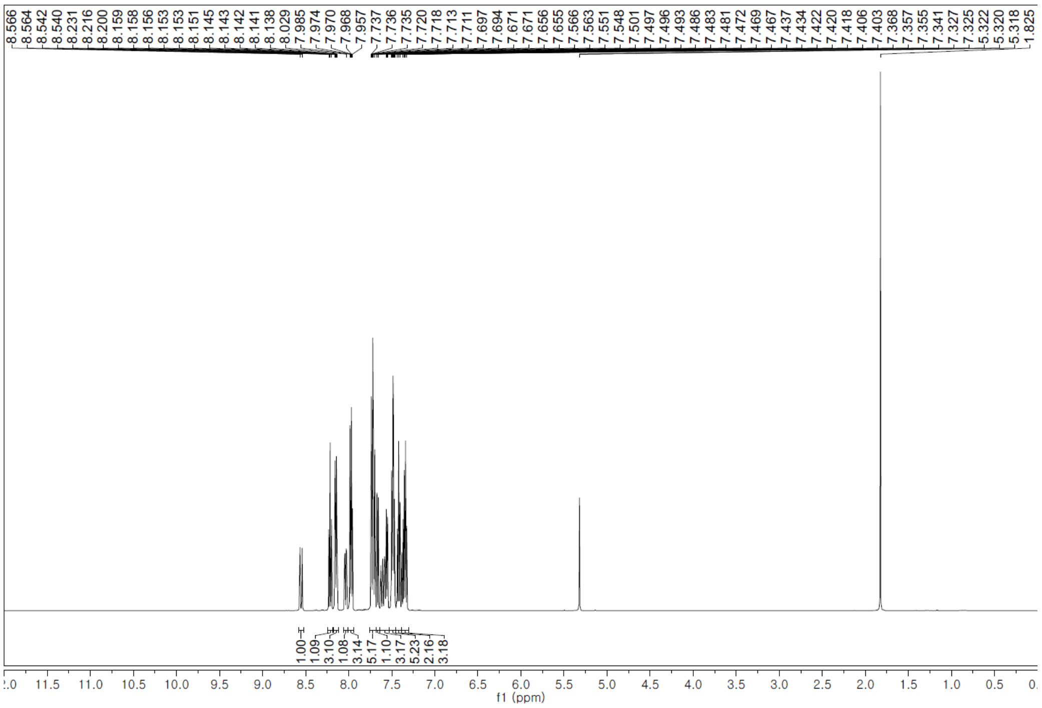

Figure S6. ^{1}H NMR spectra of **mCPyPO**

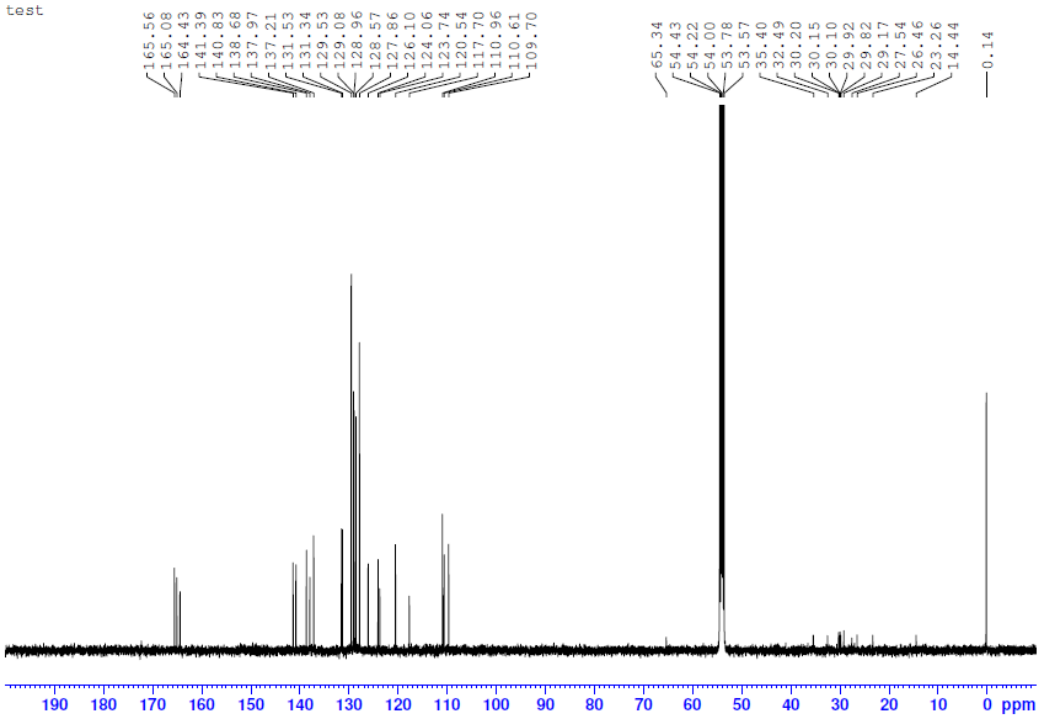

Figure S7. ^{13}C NMR spectra of **BDPyInCz**

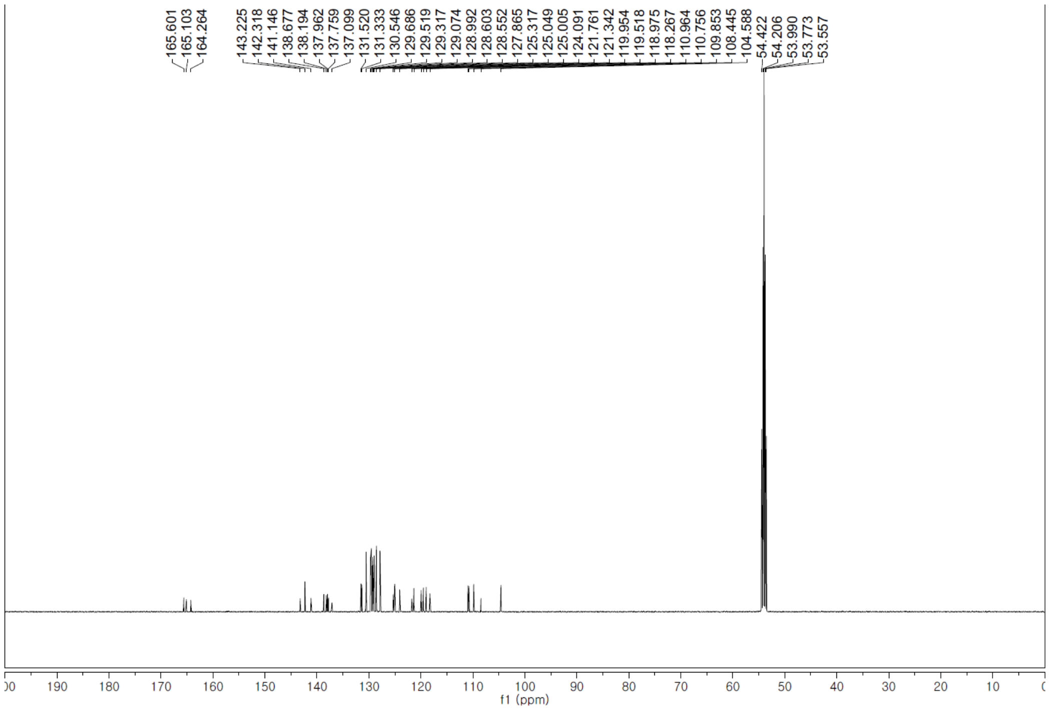

Figure S8. ^{13}C NMR spectra of **DpyInCz**

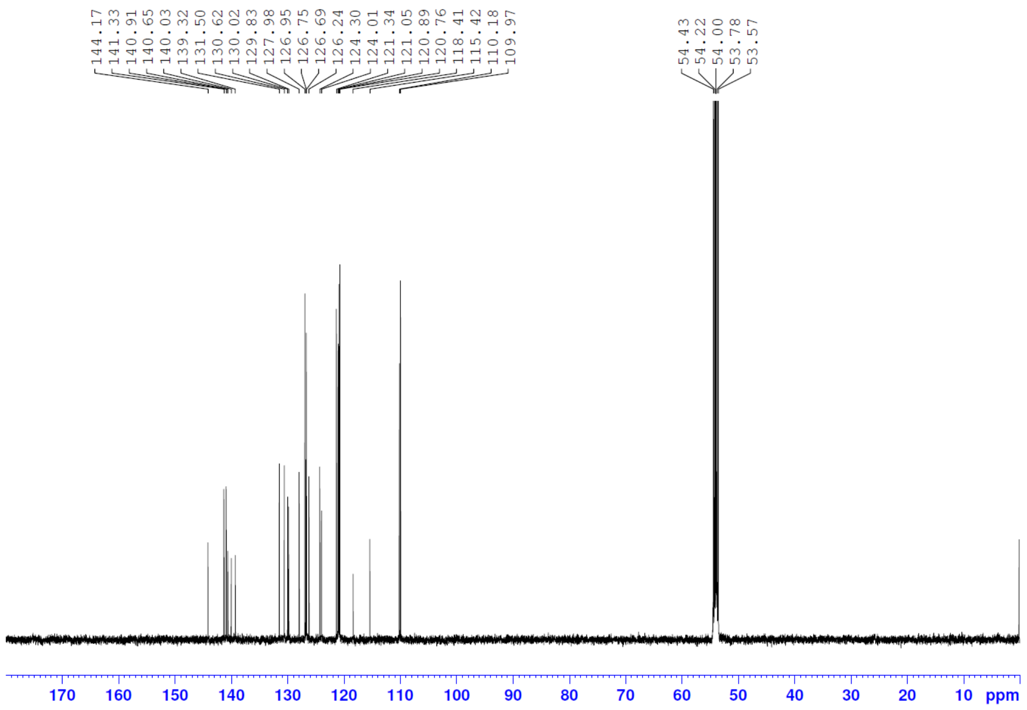

Figure S9. ^{13}C NMR spectra of **mCBP-CN**

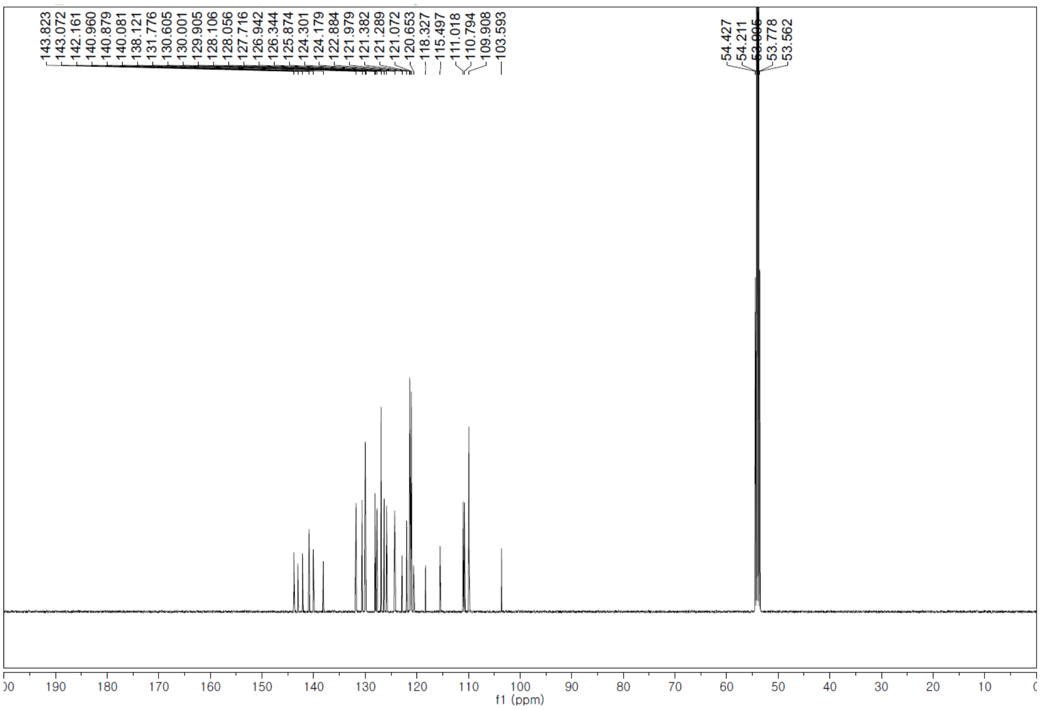

Figure S10. ^{13}C NMR spectra of **mCBP-2CN**

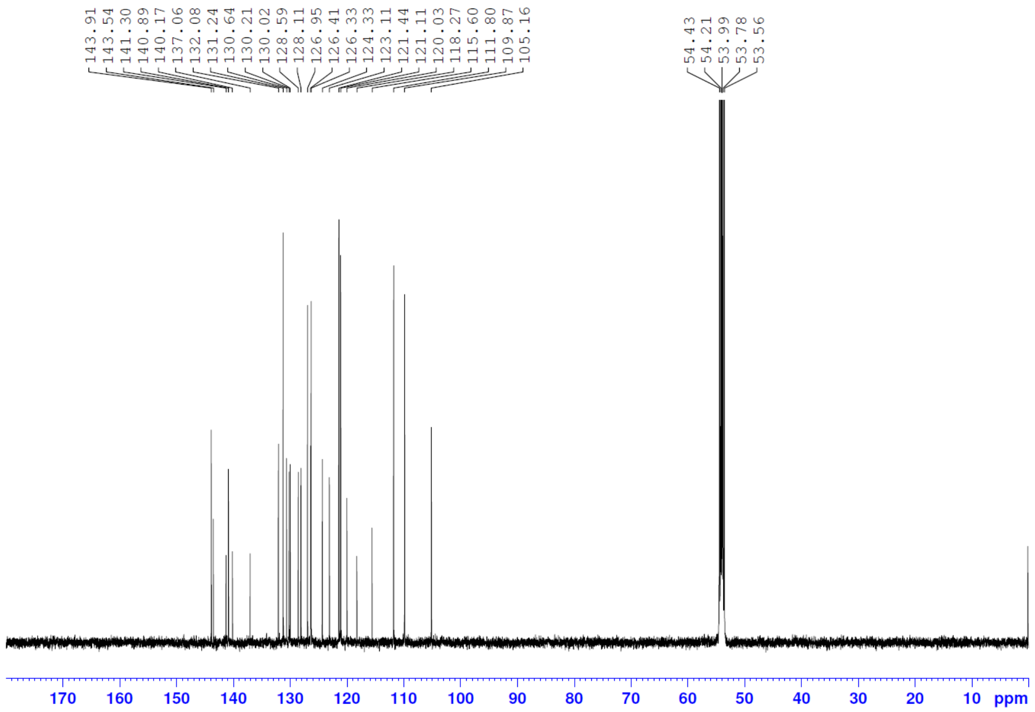

Figure S11. ^{13}C NMR spectra of **mCBP-3CN**

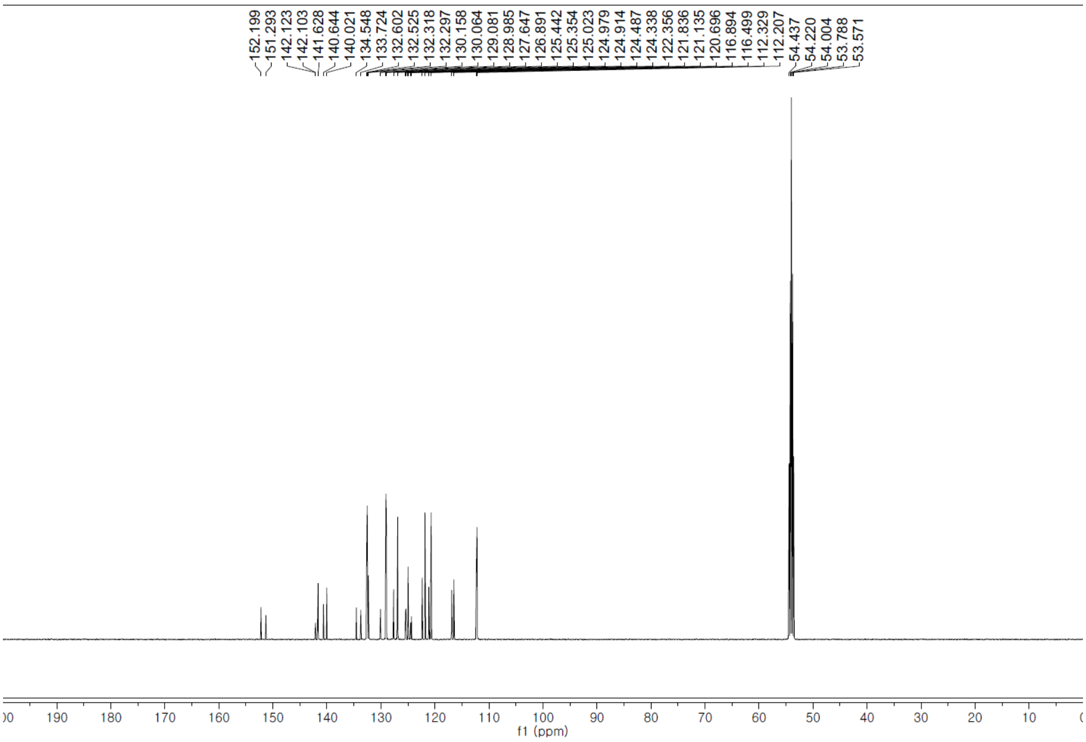

Figure S12. ^{13}C NMR spectra of **mCPyPO**

2 Photophysical properties

2.1 Methods for photophysical characterization

The Ultraviolet–Visible (UV-Vis) spectra were obtained by means of a Varian model UV-Vis-NIR spectrophotometer 5000 and the fluorescence spectra were measured on a HITACHI F7000 spectrometer for the solution states The UV-Vis absorption and solution PL emission spectra of materials were obtained from dilute toluene solution (1×10^{-5} M), while the solid PL spectra were obtained from thin films prepared by vacuum evaporation. Triplet energy values of the TADF materials were obtained from the PL spectra at 77 K using liquid nitrogen. PL quantum yield in the solution were detected with a Hamamatsu absolute PL quantum yield spectrometer C11347 (Quantaurus-QY). PL spectra and transient PL decay profiles in the solid states at ambient temperature were measured on a PicoQuant FluoTime 300 Fluorescence Lifetime spectrometer based on time-correlated single photon counting (PicoQuant, PicoHarp 300). A pulsed LED (PicoQuant, PLS 340) with an excitation wavelength of 340 nm and a single photon sensitive photomultiplier tube (PicoQuant, PMA-C) were used. The comparison between as-deposited and 3-h UV-laser-exposed films (PL stability experiment) was performed for each film using a He-Cd laser (KIMMON KOHA, IK3202R-D) at 3.5 mW with an excitation wavelength of 325 nm. The energy levels such as HOMO were measured by using cyclic voltammetry (CV). Each material was dissolved in anhydrous dichloromethane with 0.1 M tetrabutylammonium hexafluorophosphate as the electrolyte to measure the oxidation from which the HOMO energy level was estimated. A glassy carbon electron was used as the working electrode a platinum wire used as a counter electrode, and a saturated Ag/AgCl was used a reference electrode. Ferrocene was used as the standard reference. All solutions were purged with nitrogen for 10 minutes before each experiment.

2.2 Photoluminescence of BDPyInCz

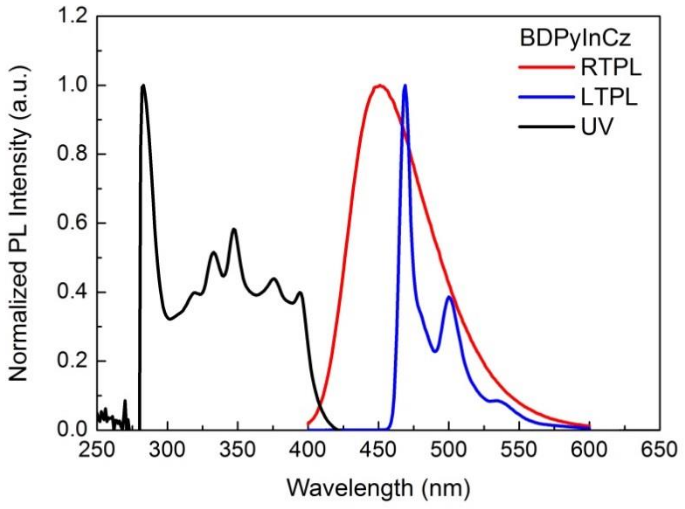

Figure S13. Absorption and photoluminescence spectra of **BDPyInCz**.

2.3 Photoluminescence of DPyInCz

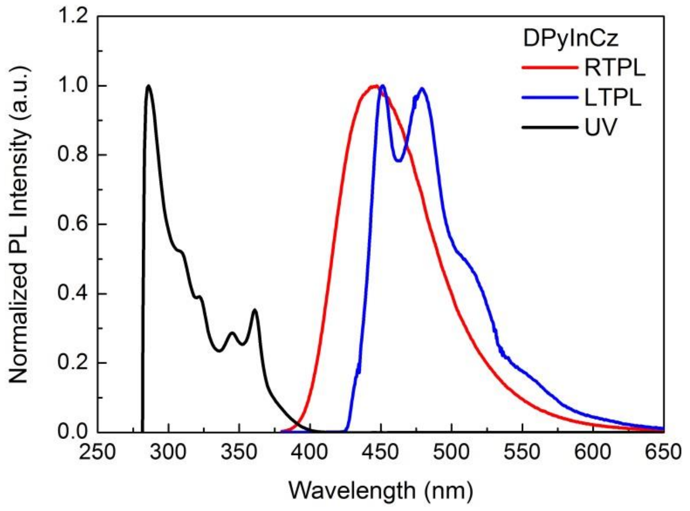

Figure S14. Absorption and photoluminescence spectra of **DPyInCz**.

2.4 Photoluminescence of mCBP-CN

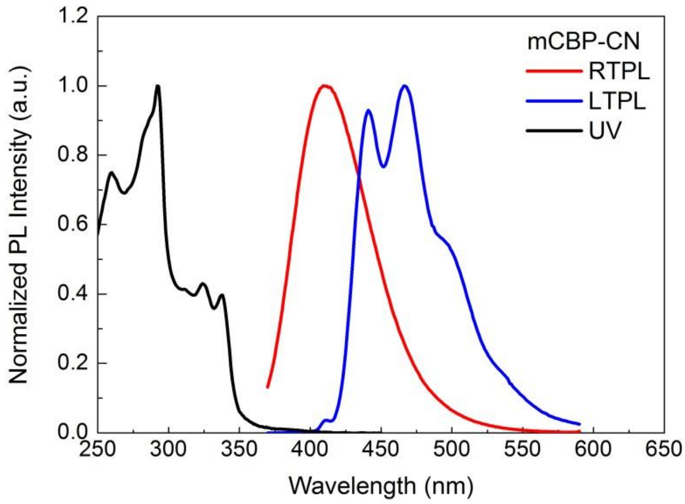

Figure S15. Absorption and photoluminescence spectra of **mCBP-CN**

2.5 Photoluminescence of mCBP-2CN

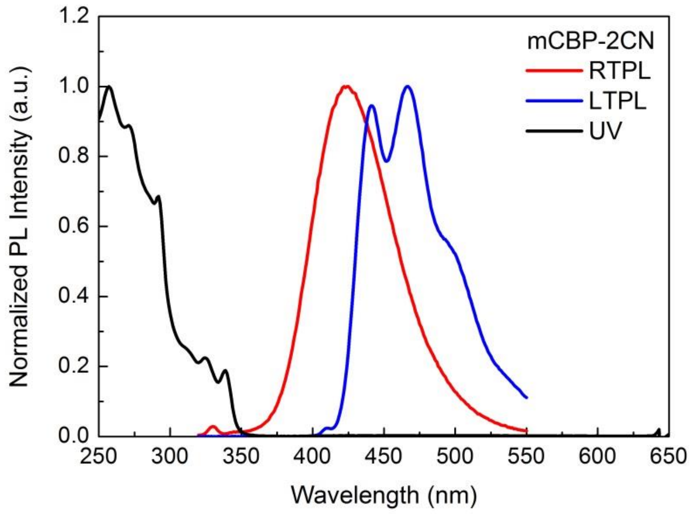

Figure S16. Absorption and photoluminescence spectra of **mCBP-2CN**.

2.6 Photoluminescence of mCBP-3CN

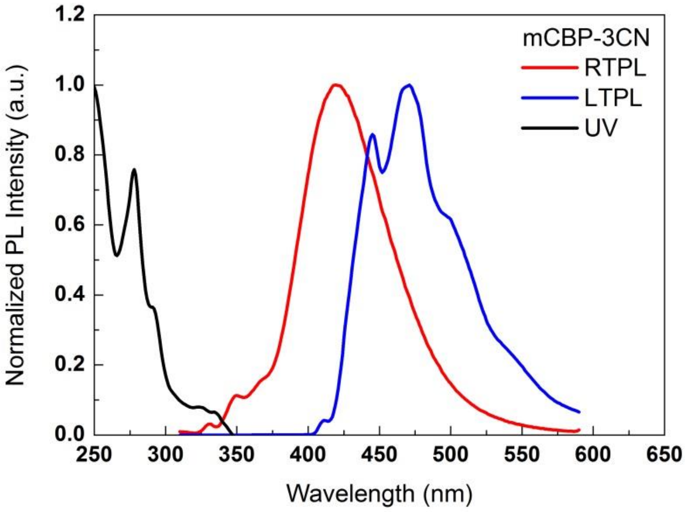

Figure S17. Absorption and photoluminescence spectra of **mCBP-3CN**.

2.7 Summary of the photophysical properties

Table S1. Calculation data of **BDPyInCz**, **DPyInCz**, **mCBP-CN**, **mCBP-2CN** and **mCBP-3CN**.

	TD-DFT				
	HOMO (eV)	LUMO (eV)	S_1 (eV)	T_1 (eV)	ΔE_{ST} (eV)
**BDPyInCz**	−5.020	−1.830	2.810	2.630	0.180
**DPyInCz**	−5.051	−1.783	2.875	2.764	0.111
**mCBP-CN**	−5.555	−1.815	3.246	3.008	-
**mCBP-2CN**	−5.779	−1.977	3.200	2.984	-
**mCBP-3CN**	−5.868	−2.138	3.135	2.949	-

All calculations were performed using the Gaussian program package[1]. The geometry in S_0 was optimized by DFT calculation. Calculations of S_1 and T_1 were performed at the TD-DFT level. Both ground and excited state calculations were performed at the B3LYP/6-31G* level.

Table S2. Measured photophysical properties of **BDPyInCz**, **DPyInCz**, **mCBP-CN**, **mCBP-2CN** and **mCBP-3CN**.

	Experiment						
	HOMO (eV)	LUMO (eV)	HOMO[a] (eV)	T_1[b] (eV)	S_1[b] (eV)	ΔE_{ST}[b] (eV)	PLQY[c] (%)
**BDPyInCz**	−5.34	−2.30	-5.80	2.64<br>*2.70*	2.75<br>*3.02*	0.11<br>*0.32*	67
**DPyInCz**	−5.42	−2.29	-5.85	2.75<br>*2.91*	2.78<br>*3.13*	0.03<br>*0.22*	30
**mCBP-CN**	−5.68	−2.12	−6.25	2.81<br>*2.94*	3.01<br>*3.34*	-	-
**mCBP-2CN**	−5.75	−2.24	−6.50	2.79<br>*2.95*	2.98<br>*3.37*	-	-
**mCBP-3CN**	−5.75	−2.22	−6.48	2.78<br>*2.97*	2.95<br>*3.34*	-	-

[a] Measured in film with a photo-electron spectrometer (RKI Instruments, AC-3)

[b] Values in *italic* correspond to those at onset properties of photoluminescence

[c] Measured in toluene under N_2

2.8 Transient photoluminescence of the films

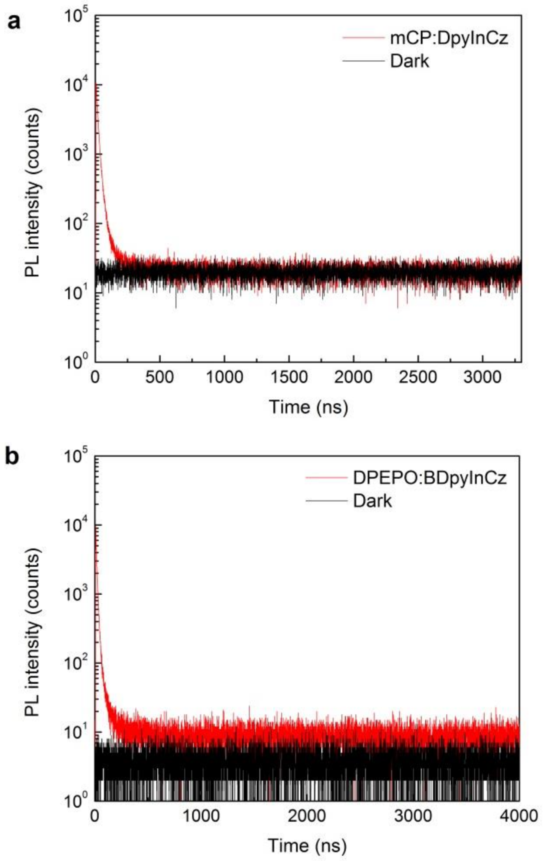

c

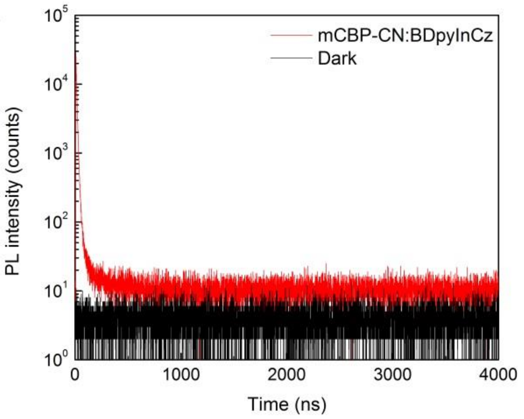
mCBP-CN:BDpyInCz
Dark
PL intensity (counts)
10^0
10^1
10^2
10^3
10^4
10^5
0
1000
2000
3000
4000
Time (ns)

d

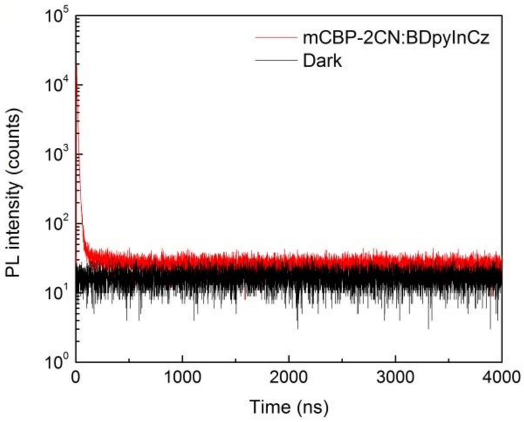
mCBP-2CN:BDpyInCz
Dark
PL intensity (counts)
10^0
10^1
10^2
10^3
10^4
10^5
0
1000
2000
3000
4000
Time (ns)

e

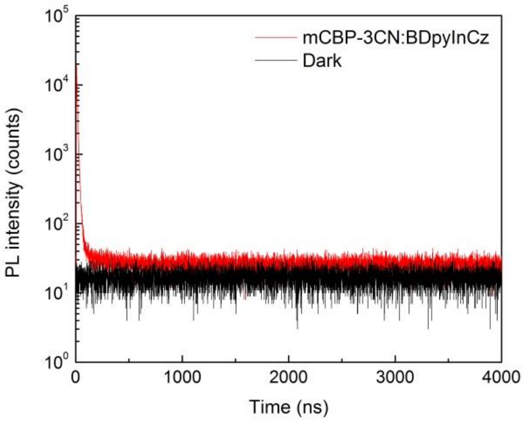
mCBP-3CN:BDpyInCz
Dark
PL intensity (counts)
10^0
10^1
10^2
10^3
10^4
10^5
0
1000
2000
3000
4000
Time (ns)

f

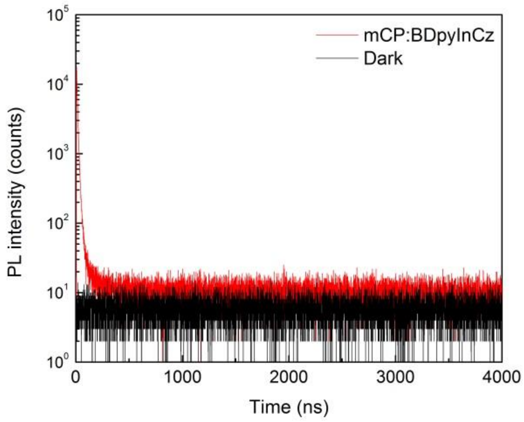
mCP:BDpyInCz
Dark
PL intensity (counts)
10^0
10^1
10^2
10^3
10^4
10^5
0
1000
2000
3000
4000
Time (ns)

Figure S18. Transient photoluminescence spectra a) **mCP:DPyInCz**, b) **DPEPO:BDPyInCz**, c) **mCBP-CN:BDPyInCz**, d) **mCBP-2CN:BDPyInCz**, e) **mCBP-2CN:BDPyInCz** and f) **mCP:BDPyInCz** in solid state (film). **DpyInCz** which shows a remarkable dual fluorescence (Fig. 4c) does not show any meaningful delayed fluorescence.

2.9 Photoluminescence stability

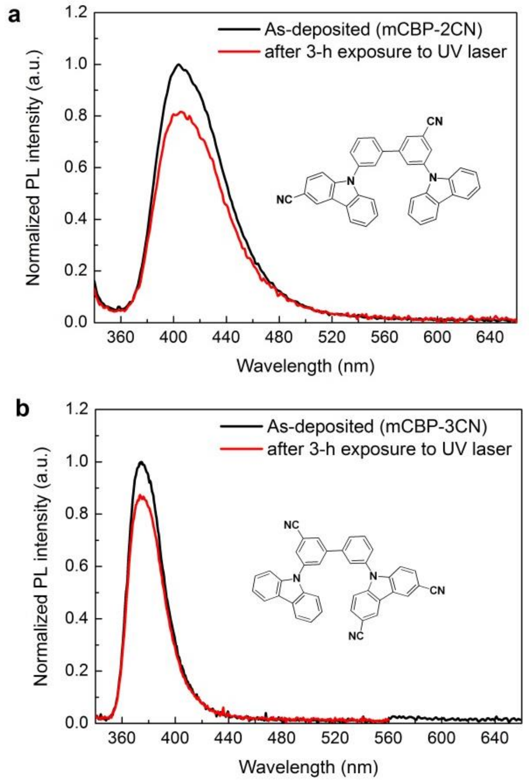

Figure S19. Comparison of PL spectra between as-deposited and 3-h UV-laser exposed films of (a) **mCBP-2CN** and (b) **mCBP-3CN**. The PL of the **mCBP-CN** (Fig. 3d), **mCBP-2CN** and **mCBP-3CN** films after 3-h exposure to UV-laser loses its peak intensity down to 0.93, 0.80 and 0.86, respectively.

3 Carrier transporting and electroluminescence properties

3.1 Device fabrication and characterization

The organic layers were consequently deposited on pre-cleaned ITO glass substrates using a thermal evaporation system with a vacuum pressure of < 1.0×10^{-6} torr. A 1-nm-thick Liq and

100-nm-thick Al (or Ag) were deposited as a cathode by thermal evaporation. The deposition rates of the organic and metal layers were about 0.1 nm s^{-1} and 0.5 nm s^{-1}, respectively. That of the Liq layer was about 0.01 nm s^{-2}. The active device area of 4 mm^2 was defined by the overlapped area of the ITO and Al electrodes. The HOD structure is ITO/HAT-CN(10 nm)/NPB(50 nm)/test material(30 nm)/NPB(10 nm)/Al. The EOD structure is ITO/Ag/DBFPO:Liq(10 nm)/test material(30 nm)/DBFPO:Liq(30 nm)/Liq(1 nm)/Ag. Current, voltage and luminance of the OLEDs were measured with a Keithley 2400 Source-Meter and PR-650 spectroradiometer. Lifespan measurement of the OLEDs was performed at a constant current mode.

3.2 Current density-voltage curves of HODs and EODs for various hosts

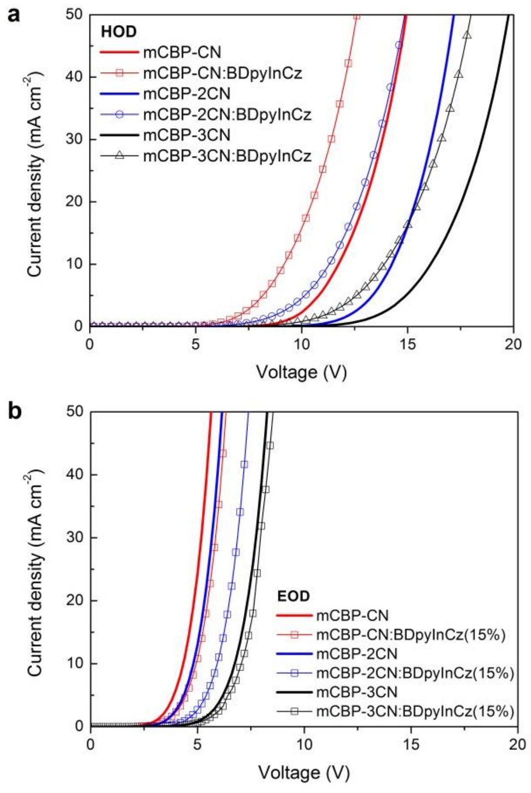

Figure S20. Current density-voltage curves of a) HODs and b) EODs for **mCBP-CN:BDPyInCz**, **mCBP-2CN:BDPyInCz** and **mCBP-3CN:BDPyInCz**. **mCBP-CN** exhibits best ability in charge transporting among them and it is clear that the HT-type BDpyInCz improves the hole transporting property for all cases as expected.

3.3 Stability of HODs and EODs for various hosts

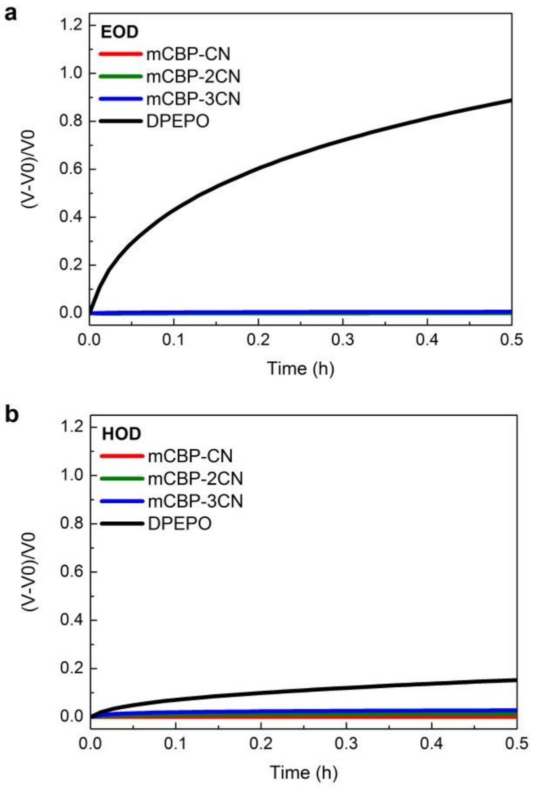

Figure S21. Change of the driving voltages of a) EODs and b) HODs of **mCBP-CN**, **mCBP-2CN**, **mCBP-3CN** and **DPEPO** keeping the driving current constant. The EOD of the three new hosts are much more stable than that of DPEPO but no difference is observed between the three. The HOD of **mCBP-CN** among the three new hosts is however most stable but the difference is not so much remarkable though.

3.4 Electroluminescence spectra of BDPyInCz in mCBP-(n)CN matrices

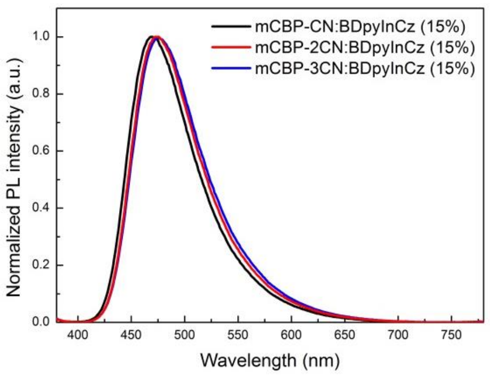

Figure S14. EL spectra of **BDPyInCz** in **mCBP-(n)CN**-based host matrices with 15% doping concentrations.

3.5 Electroluminescence performances

Table S3. Device performances of BDpyInCz:mCBP-(n)CN-based TADF OLEDs. The same device structure was utilized for comparison of the three hosts.

	V_d* (V)	CIEx	CIEy	λ_{EL}† (nm)	Quantum Efficiency (%)			LT80 (h)
					10 cd m^{-2}	100 cd m^{-2}	500 cd m^{-2}	
mCBP:BDpyInCz (15%)	7.64	0.160	0.201	464	7.9	7.3	5.7	1.3
mCBP-CN:BDpyInCz (15%)	4.78	0.167	0.237	470	13.0	10.6	8.0	16.0
mCBP-2CN:BDpyInCz (15%)	4.45	0.173	0.270	474	11.4	9.4	7.3	7.6
mCBP-3CN:BDpyInCz (15%)	4.74	0.176	0.286	475	10.8	8.5	6.4	8.9

* Driving voltage at a luminance of 500 cd m^{-2}.

† Wavelength of the EL peak.
